# Transcriptome Analysis Reveals Cr(VI) Adaptation Mechanisms in *Klebsiella* sp. Strain AqSCr

**DOI:** 10.3389/fmicb.2021.656589

**Published:** 2021-05-27

**Authors:** Paloma Lara, Leticia Vega-Alvarado, Diana X. Sahonero-Canavesi, Michel Koenen, Laura Villanueva, Fernando Riveros-Mckay, Enrique Morett, Katy Juárez

**Affiliations:** ^1^Departamento de Ingeniería Celular y Biocatálisis, Instituto de Biotecnología, Universidad Nacional Autónoma de México, Cuernavaca, Mexico; ^2^Instituto de Ciencias Aplicadas y Tecnología, Universidad Nacional Autónoma de México, Ciudad de México, Mexico; ^3^Department of Marine Microbiology and Biogeochemistry (MMB), NIOZ Royal Netherlands Institute for Sea Research, Texel, Netherlands; ^4^Faculty of Geosciences, Department of Earth Sciences, Utrecht University, Utrecht, Netherlands

**Keywords:** *Klebsiella* sp. AqSCr, transcriptome, chromate resistance, chromate reduction, fatty acid desaturase

## Abstract

*Klebsiella* sp. strain AqSCr, isolated from Cr(VI)-polluted groundwater, reduces Cr(VI) both aerobically and anaerobically and resists up 34 mM Cr(VI); this resistance is independent of the ChrA efflux transporter. In this study, we report the whole genome sequence and the transcriptional profile by RNA-Seq of strain AqSCr under Cr(VI)-adapted conditions and found 255 upregulated and 240 downregulated genes compared to controls without Cr(VI) supplementation. Genes differentially transcribed were mostly associated with oxidative stress response, DNA repair and replication, sulfur starvation response, envelope-osmotic stress response, fatty acid (FA) metabolism, ribosomal subunits, and energy metabolism. Among them, genes not previously associated with chromium resistance, for example, *cybB*, encoding a putative superoxide oxidase (SOO), *gltA2*, encoding an alternative citrate synthase, and *des*, encoding a FA desaturase, were upregulated. The *sodA* gene encoding a manganese superoxide dismutase was upregulated in the presence of Cr(VI), whereas *sodB* encoding an iron superoxide dismutase was downregulated. Cr(VI) resistance mechanisms in strain AqSCr seem to be orchestrated by the alternative sigma factors *fecl*, *rpoE*, and *rpoS* (all of them upregulated). Membrane lipid analysis of the Cr(IV)-adapted strain showed a lower proportion of unsaturated lipids with respect to the control, which we hypothesized could result from unsaturated lipid peroxidation followed by degradation, together with *de novo* synthesis mediated by the upregulated FA desaturase-encoding gene, *des*. This report helps to elucidate both Cr(VI) toxicity targets and global bacterial response to Cr(VI).

## Introduction

Chromium is widely used in several industrial processes; unfortunately, its inadequate waste management has resulted in serious soil and aquifer pollution problems, mainly for the hexavalent oxidation state [Cr(VI)] (Shi and Dalal, 1990; Viti et al., 2014). Because of its high solubility, strong oxidizing power, mutagenicity, and bioavailability through the sulfate transport system, Cr(VI) is a highly toxic pollutant (Pereira et al., 2008). Once inside the cell, Cr(VI) is reduced to Cr(III) through the short-lived intermediates Cr(V) and Cr(IV), which promote generation of reactive oxygen species (ROS) (Shi and Dalal, 1990; Jones et al., 1991; Shi et al., 1991; Clementino et al., 2018; Wakeel et al., 2020), DNA adducts, DNA inter- and intra-strand crosslinks and breaks, DNA–protein crosslinks, base oxidation, and abasic sites (Wedrychowski et al., 1986; Kortenkamp et al., 1996; Casadevall et al., 1999; Slade et al., 2005; Santos-Escobar et al., 2019). Due to its toxicity, bioremediation strategies have been focused on Cr(VI) reduction to Cr(III) (Marsh and McInerney, 2001; Lara et al., 2017; Lacalle et al., 2020), which is less toxic and less soluble at neutral pH, resulting in its immobilization as chromium(III) hydroxide precipitates in soils and aquatic environments (Sass and Rai, 1987; Lacalle et al., 2020). Several phylogenetically and metabolically diverse bacteria have been described to be able to reduce Cr(VI) to Cr(III) (Narayani and Shetty, 2013). This microbial activity could be exploited as a safe and cost-effective remediation technology, as an alternative to expensive traditional physicochemical approaches (Xiao et al., 2014). To develop effective methodologies, a comprehensive understanding of the bacterial adaptation and responses to metals and biotransformation processes are essential. Microbial resistance to Cr(VI) has been associated principally with a chromate transporter that pumps the metal out of the cell. However, in recent years, reports of transcriptional and proteomic bacterial global response to chromium have indicated a more complex system related to resistance and reduction of Cr(VI). In this study, we report the characterization by traditional and omics approaches of new Cr(VI)-reducing *Klebsiella* sp. strain AqSCr, isolated from chromate-contaminated groundwater, with exceptional metabolic capabilities. A primary rationale for studying this strain was to characterize indigenous organisms adapted to the physicochemical characteristics of the highly polluted aquifer from which it was isolated. For strain AqSCr, resistance to Cr(VI) was higher at alkaline pH, and the deletion of a *chrA* gene encoding a putative chromate transporter did not affect Cr(VI) resistance. Subsequent subculturing of strain AqSCr in the presence of Cr(VI) leads to metabolic adaptation, resulting in a shorter lag phase. The global transcriptional profile in cells adapted to Cr(VI) compared with control conditions without Cr(VI), show that differentially transcribed genes were mainly involved in the response to oxidative stress, the response to osmotic stress, repair and DNA replication, uptake of sulfur, phosphate, molybdate, iron, and manganese, response to the cell envelope stress, ribosomal functions, and energy metabolism.

## Materials and Methods

### Isolation of *Klebsiella* sp. Strain AqSCr

*Klebsiella* sp. strain AqSCr was isolated from groundwater of a Cr(VI)-polluted industrial landfill located in León, Guanajuato, Mexico (21° 04′ 2″ N, 101° 79′ 10″ W). First, Cr(VI)-resistant bacteria from groundwater were enriched in mineral medium with acetate-fumarate (NBAF) (Coppi et al., 2001) without resazurin and cysteine, supplemented with 0.05% yeast extract and 2 mM Cr(VI), at 30°C under anoxic conditions. After serial dilution, cells were plated on solid NBAF medium and grown anaerobically, in an anaerobic chamber (Model 1025, Forma Scientific, Marietta, OH, United States) with an atmosphere of N_2_:H_2_:CO_2_ 85:10:5. Finally, strain AqSCr was purified by repeated subculturing on agar plates.

### Culture Media

Strain AqSCr was grown under aerobic conditions at 30°C at 250 rpm in LB medium (Bertani, 1951) and in Cit-Na medium containing HOC(COONa) (CH_2_COONa)_2_⋅2H_2_O 15.146 g/L, NH_4_Cl 2.5 g/L, MgSO_4_⋅7H_2_O 1.5 g/L, KCl 0.1 g/L, NaH_2_PO_4_⋅H_2_O 0.06 g/L, and supplemented with casamino acids at 0.2% after sterilization. For alkaline conditions, pH was adjusted with Na_2_CO_3_⋅H_2_O.

For anaerobic conditions, strain AqSCr was grown at 30°C without agitation in LB, NBAF (Coppi et al., 2001), and sodium citrate media. Anaerobic sodium citrate medium contains HOC(COONa) (CH_2_COONa)_2_⋅2H_2_O 15.146 g/L, Na_2_CO_3_⋅H_2_O 0.5 g/L, NH_4_Cl 2.5 g/L, MgSO_4_⋅7H_2_O 1.5 g/L, KCl 0.1 g/L, NaH_2_PO_4_⋅H_2_O 0.06 g/L, and casamino acids at 0.2% added after sterilization.

Tubes with media were flushed with N_2_ and CO_2_ 80:20, bubbling 8 min the liquid phase and flushing 8 min the headspace to remove oxygen (for each 10 ml), sealed with rubber stoppers and metal crimp-tops and autoclaved. Inoculation, supplementation, and sampling were carried out with syringes flushed with N_2_:CO_2_ 80:20, to preserve anaerobic conditions. In the case of LB medium, only N_2_ was used for O_2_ displacement.

### Genome Sequencing

Genomic DNA from strain AqSCr was extracted using a MasterPure DNA purification kit (Epicenter, Madison, WI, United States). Paired-end and mate-pair libraries were sequenced using an Illumina GAIIX at the University Massive DNA Sequence Unit, Instituto de Biotecnología, Universidad Nacional Autónoma de México (UUSMD-IBT, UNAM). Reads were corrected and assembled using Spades 3.0.0 (Bankevich et al., 2012). The DNA sequence was deposited in GenBank with the accession number MJDM00000000 (BioSample SAMN05730075). Functional annotations of the predicted genes were performed using the RAST server (Rapid Annotation using Subsystem Technology) (Aziz et al., 2008). Genome analyses were performed using the SEED server http://rast.nmpdr.org/seedviewer.cgi. Comparison of amino acid sequence of putative chromate reductases was performed with ClustalW (Larkin et al., 2007).

The chromosome sequence of strain AqSCr was compared to the closest organism genome (based on 16S rRNA gene analysis). Analysis was performed using nucmer, delta-filter, and show-coords programs from MUMmer 3.23 package (Kurtz et al., 2004). Each contig was aligned to the reference using nucmer, repetitive alignments were filtered out, and the best alignment for each assembled contig was selected using delta-filter (-q) program; the coordinates of the resulting alignment were obtained by show-coords program. Finally, the sum of the alignment lengths for each contig was calculated to obtain the percentage of coverage of the reference in the assembly.

### Chromate Resistance

Strain AqSCr was cultivated in aerobic liquid LB media at pH 7, 8, and 9 with 0.5–34 mM Cr(VI) and incubated for 24 h at 30°C at 250 rpm. Then, serial 10-fold dilutions were spotted onto solid LB plates and incubated at 30°C for 48 h to determine colony-forming units (CFUs).

### Metabolic Adaptation of Strain AqSCr to Cr(VI)

Strain AqSCr was aerobically subcultured twice in LB medium at pH 8 with and without 11 mM Cr(VI). These cultures were used to inoculate fresh LB medium (pH 8), containing 11 mM Cr(VI) to monitor growth at 30°C and 250 rpm. CFUs in the cultures were determined at 0, 3, 6, 9, 25, and 27 h of incubation by spotting serial 10-fold dilutions on solid LB agar plates. Subsequently, plates were incubated at 30°C for 48 h.

### Chromate Reductase Activity

Twenty-four-hour cultures were harvested by centrifugation at 4,500 × *g* for 20 min at 4°C, washed twice with 10 mM Tris–HCl (pH 7), and lysed with a French press (model FA078A with 20 K pressure cell; Thermo Spectronic, Rochester, NY, United States) at 965.3 MPa. The cell lysate was centrifuged for 15 min at 1,460 × *g* to remove cell debris and later ultracentrifuged for 143 min at 45,000 rpm at 4°C in a Beckman SW 50.1 rotor. The supernatant was transferred to an anaerobic tube and designated as the soluble fraction. The pellet was suspended in 10 mM Tris–HCl (pH 7) and designated as the membrane fraction. One milliliter of the soluble fraction was transferred into ultrafiltration tubes (Amicon Ultra 10K and 50K devices; Millipore, Germany) and centrifuged at 11,000 × *g* at 4°C for 10 min to retain proteins.

Chromate reductase activity was assayed anaerobically at 30°C; the reaction mixture contained 100 μl of cell extract, 100 μl of 4.4 mM NADH as the electron donor, and 2 ml of 0.025 mM K_2_Cr_2_O_7_ as the electron acceptor in N_2_-saturated 10 mM Tris–HCl (pH 7) (total reaction volume = 2.2 ml). The reaction was sampled periodically and the Cr(VI) concentration was measured as described below.

### Analytical Methods

Hexavalent chromium Cr(VI) was determined colorimetrically at 540 nm by diphenyl carbazide reaction in acid solution (American Public Health Association, 1999). Divalent iron was quantified by a colorimetric method with FerroZine^TM^ Iron Reagent [3-(2-Pyridyl)-5,6-diphenyl-1,2,4-triazine-*p*,*p*′-disulfonic acid monosodium salt] (Viollier et al., 2000). Citrate was quantified at 210 nm in an Agilent series 1100 HPLC (Agilent Technologies, Inc., Albany, NY, United States) with an Aminex HPX-87H (Bio-Rad, Hercules, CA, United States) column. Protein concentration of cellular extracts was quantified by the Bradford method (Bradford, 1970).

### Creation of Deletion Mutants of *Klebsiella* sp. Strain AqSCr

Genes encoding putative soluble chromate reductases (Supplementary Tables 1, 2) and the ChrA transporter were deleted from the wild-type strain Klebsiella sp. strain AqSCr by double homologous recombination with a modified protocol of Datsenko and Wanner (2000), using primers indicated in Supplementary Table 2. Plasmid vectors pIJ790ΩSp and pCP20-ΩSp, which contained a spectinomycin-resistance cassette, were used instead of the original vectors pIJ790 and pCP20 (which contain an ampicillin-resistance gene). The recombinant PCR products consisted of the kanamycin resistance cassette (flanked by FRT sequence) from the pKD13 plasmid (Datsenko and Wanner, 2000), flanked by approximately 400 bp from the region flanking the target gene, obtained as described by Wei et al. (2012). After selection, the kanamycin-resistance cassette was eliminated, and gene deletion was confirmed by PCR. To generate the multiple mutants, the protocol was repeated for each gene.

### Transmission Electron Microscopy

Cells grown aerobically in LB medium (pH 8) containing 11 mM Cr(VI) were fixed in paraformaldehyde 4% and glutaraldehyde 3%, washed with 0.16 M sodium cacodylate at pH 7.2, and dehydrated with ethanol 70% to absolute ethanol, and subsequently were immersed in propylene oxide with Epon to obtain 60-nm slices. The ultrathin sections were then treated with or without 3% uranyl acetate and examined under a Zeiss LIBRA 120 transmission electron microscope (Zeiss, Oberkochen, Germany) operated at 120 kV.

### RNA Extraction and Sequencing

Cells of strain AqSCr were subcultured twice in LB medium (pH 8) aerobically with [11 mM Cr(VI)] and without (control) Cr(VI) with an optical density (O.D.) 600 nm of 0.64 ± 0.05 and 1.05 ± 0.05, respectively (i.e., exponential growth phase). Cultures were immediately (<10 s) added with RNAlater^®^ (Thermo Fisher Scientific) and incubated on ice for 30 min, after which they were centrifuged 20 min at 1,460 × *g*, supernatants were discarded, and the cell pellets were preserved at −65°C.

Total RNAs were extracted from each sample using the RNeasy Mini Kit (QIAGEN). Then, total RNA was digested with DNase I (Thermo Fisher Scientific) and consecutively digested with the enzyme Terminator^TM^ 5′-Phosphate-Dependent Exonuclease (Epicenter) to eliminate ribosomal RNA. Experiments were conducted in triplicate. Finally, a total of six paired-end libraries were sequenced using an Illumina GAIIX at the University Massive DNA Sequence Unit, Instituto de Biotecnología, Universidad Nacional Autónoma de México.

### RNA-Seq Data Processing and Analysis

Raw data were trimmed, removing short reads, adapter–dimer reads, reads with an N ratio (number of unknown nucleotides/number of total nucleotides) greater than 5%, and reads with more than 20% low-quality nucleotides (Phred quality score < 10). Reads were mapped onto the genes of *Klebsiella* sp. strain AqSCr, using Bowtie2 (version 2.3.4.3) software (Langmead and Salzberg, 2012) and counted using coverageBed function from BedTools2 (version 2.27.1) (Quinlan and Hall, 2010).

The differential transcription analysis was performed using four different Bioconductor (Gentleman et al., 2004) packages: DESeq2 (Love et al., 2014), NOISeq (Tarazona et al., 2015), edgeR (Robinson et al., 2010), and limma-Von (Ritchie et al., 2015). Ten counts per million (CPM) and a |log_2_(fold change, FC)| ≥ 1.5 with a false discovery rate (FDR) or adjusted *p* value (*p*-adj) ≤ 0.01 were used as the cutoff; genes detected by at least three methods were declared differentially transcribed. These analyses were performed using the Integrated Differential Expression Analysis MultiEXperiment website^1^ (Jiménez-Jacinto et al., 2019). The amino acid sequences of proteins encoded by differentially transcribed genes were subject to KO assignment (KEGG orthology) for functional categorization and metabolic pathway reconstruction with GhostKoala of KEGG^2^. The RNA-Seq data were deposited in NCBI’s Gene expression Omnibus (Edgar et al., 2002), accessible through GEO series accession number GSE160968. Genes associated with functional categories previously linked to metal resistance were manually curated; comparing the associated KO with the automatic annotation and amino acid sequences from genes without assigned KO were compared to putative homologous using ClustalW (Larkin et al., 2007).

### RNA-Seq Validation Using Reverse Transcription Quantitative PCR

RNA-Seq results were validated by reverse transcription quantitative PCR (RT-qPCR) of 13 selected genes. The gene-specific oligonucleotides used in this study are shown in Supplementary Table 3, and their specificity was confirmed by melting curve analysis. The *alaRS* (BHE81_13165) gene (coding the Alanyl-tRNA synthetase) was used as the internal reference. For cDNA synthesis, 1 μg of RNA was reverse transcribed using the RevertAid H Minus Reverse Transcriptase (ThermoFisher Scientific^®^). The cDNAs were quantitatively analyzed with a real Quant Studio 12K Flex System (Life Technologies, Foster City, CA, United States) using the kit Maxima SYBR Green/ROX qPCR Master Mix (2X) (ThermoFisher Scientific^®^). The following program was used: 95°C for 10 min, followed by 40 cycles of 95°C for 15 s, 60°C for 60 s. Melting temperature-determining dissociation steps were performed at 95°C, 40°C for 30 s. Finally, the relative transcription ratios were calculated by the ΔΔCT method (Fleige et al., 2006) as the relative quantity of the target gene transcript from cells grown in LB medium (pH 8) with 11 mM Cr(VI) divided by the relative quantity of the target gene transcript from cells grown in LB pH 8 without Cr(VI).

### Lipid Analysis

Strain AqSCr was grown under the same conditions used for RNA-Seq analysis in triplicate with and without Cr(VI). For fatty acid (FA) analysis, the freeze-dried biomass was hydrolyzed by refluxing with HCl/MeOH (1.5 N) for 3 h; subsequently, the pH was adjusted with KOH to five and extracted with dichloromethane. The resulting extracts were methylated with diazomethane in diethyl ether to transform FAs into fatty acid methyl esters (FAMEs) and the hydroxyl groups were silylated using N, O-bis(trimethylsilyl)trifluoroacetamide (BSTFA) and pyridine. Gas chromatography (GC) and GC-MS analysis were performed with similar conditions as in Bale et al. (2019). The FAME compounds were identified based on literature data and library mass spectra.

To assess whether the lipid changes observed were statistically significant, a two-way ANOVA with Sidak’s multiple comparisons test was conducted on the mean of each FAME compound measured in triplicate. A *p* value < 0.05 was considered statistically significant. Data are presented as mean ± standard error of the mean. The analysis was conducted in triplicate for each control [no Cr(VI)] or with 11 mM Cr(VI). The analysis was conducted using GraphPad Prism Software version 9.02 (GraphPad Software, San Diego, CA, United States)^3^.

## Results and Discussion

### Isolation and Draft Genome Sequencing of *Klebsiella* sp. Strain AqSCr

*Klebsiella* sp. strain AqSCr was isolated from a chromium contaminated aquifer located in León Gto. México. The genome of *Klebsiella* sp. strain AqSCr was sequenced using the Illumina GAIIX platform. The estimated genome size was 5,627,880 base pairs (bp), with an N90 of 112,278 and an L50 of 1. Assembly into 55 contigs, 10 of which covered the chromosomal DNA sequence with an average GC content of 56.6% (MJDM0100001.1: MJDM01000010.1) and contained 4,977 predicted protein-coding DNA sequences (CDSs), while the other 45 contigs correspond to plasmid DNA (MJDM01000011.1: MJDM01000055.1) with an average GC content of 50.54% and contain 427 predicted protein- CDSs. The closest sequenced genome to strain AqSCr is *Klebsiella pneumoniae* NTUH-K2044, with 92% identity over 85% coverage. The orthologs of the *chrA* (BHE81_07815) and *chrB* (BHE81_07815) genes, which encode a putative chromate efflux pump and its transcriptional regulator, respectively, were located on chromosome (contig4: MJDM01000005), although they are normally found in extrachromosomal elements. Also, six genes encoding putative homologs of soluble Cr(VI) reductases, and three genes encoding membrane-bound Cr(VI) reductases were found (Supplementary Table 1). The putative ChrA protein has 456 amino acids (aa) and belongs to the subfamily of long-chain chromate ion transporters (LCHR). Both genes *chrA* and *chrB* are commonly located in plasmids and, in some cases, in transposons but also have been found chromosomally located as in strain AqSCr (Branco et al., 2008; Caballero-Flores et al., 2012; Baaziz et al., 2017).

### Cr(VI) Resistance of *Klebsiella* sp. Strain AqSCr

**FIGURE 1:**
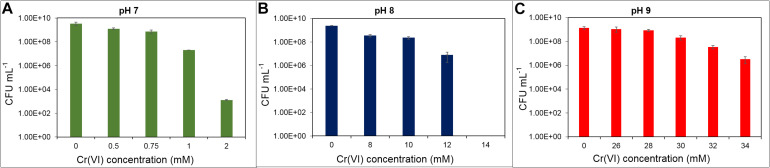
Cr(VI) resistance as indicated by growth of *Klebsiella* sp. strain AqSCr in LB medium at different pH. **(A)** pH 7, **(B)** pH 8, and **(C)** pH 9.

Cr(VI) resistance has been reported in different bacteria, ranging from 180 μM in a *Bacillus brevis* strain to 600 mM in *Nesterenkonia* sp. strain MF-2 (Amoozegar et al., 2007; Verma et al., 2009). Those differences are due to the intrinsic microbial metabolic properties and to the growth culture media used (El Baz et al., 2015). In *Shewanella oneidensis* strain MR-1, the Cr(VI) resistance limit was 2 mM in LB medium at pH 7 (Brown et al., 2006). Within the genus *Klebsiella*, chromate resistance also varies over a wide range of concentrations; for example, in *K. ornithinolytica* strain 1P and *K. oxytoca* strain P2, growth is inhibited by 0.5 and 0.75 mM Cr(VI), respectively (Garavaglia et al., 2010). Toroglu et al. (2009) also reported a strain of *Klebsiella* sp. sensitive to 1 mM Cr(VI), while *Klebsiella* sp. strain PB6, isolated from a contaminated soil, can cope with concentrations up to 10.7 mM (Wani and Omozele, 2015). Therefore, we tested the ability of the *Klebsiella* sp. strain AqSCr to grow in the presence of increasing concentrations of Cr(VI) in aerobic liquid LB media at pH 7, 8, and 9 (Figures 1A–C). The highest tolerated concentration was 34 mM at pH 9, while at pH 7, it was only 2 mM Cr(VI).

To determine the contribution of the efflux transporter *chrA* gene product (1,468 bp) to the Cr(VI) resistance, a region of 1,242 bp of this gene was deleted as described in *Materials and Methods*. The resulting mutant strain was grown with 1 and 3 mM of Cr(VI) with no effect on its growth (Supplementary Figure 1), pointing out that other mechanisms must be involved in the resistance to Cr(VI).

### Cr(VI) Reduction by *Klebsiella* sp. Strain AqSCr

**FIGURE 2:**
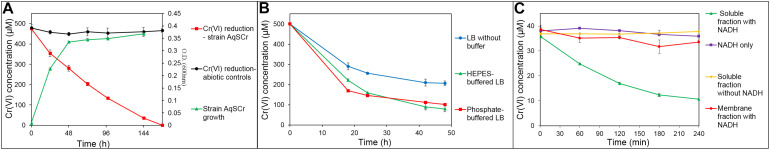
Cr(VI) reduction by *Klebsiella* sp. strain AqSCr. **(A)** Anaerobic chromate reduction in sodium citrate medium. **(B)** Aerobic chromate reduction in unbuffered LB medium, phosphate-buffered LB medium, and HEPES-buffered LB medium. **(C)** *In vitro* chromate reductase activity in soluble and membrane fractions of *Klebsiella* sp. strain AqSCr with and without NADH.

Cr(VI) reduction was evaluated in citrate basal medium and in LB medium, both under aerobic and anaerobic conditions. In anaerobic citrate basal medium (pH 7, buffered with carbonates) with 500 μM Cr(VI), complete reduction was observed in 168 h (Figure 2A). To our knowledge, this is the first report of anaerobic chromate reduction by a *Klebsiella* strain. Strain AqSCr was unable to reduce Cr(VI) in citrate basal media in aerobic conditions (data not shown).

Cr(VI) reduction in bacteria is limited by several factors, including intrinsic characteristics of the microorganism, culture parameters, and media formulation (Ohtake et al., 1990; Mabrouk, 2008), and different reduction mechanisms can act under different culture conditions in the same microorganism (Li and Krumholz, 2009). In *Enterobacter cloacae* strain HO1, the presence of specific amino acids, such as aspartate, enhances Cr(VI) reduction (Ohtake et al., 1990). Thus, the lack of reduction in aerobic citrate medium by strain AqSCr can be the result of fast consumption or low availability of specific compounds required for this process, such as amino acids.

During aerobic growth in LB medium, pH increase of the medium was observed (Supplementary Figure 2); therefore, aerobic reduction of Cr(VI) was analyzed in LB medium (pH 7) with and without HEPES or phosphate buffer. Reduction was faster with both buffered media (Figure 2B). In the *Thermoanaerobacter*-like strain BSB-33, less Cr(VI) reduction at alkaline pH compared to neutral conditions has been previously reported (with reduction decreasing in the order, pH 7 > pH 7.5 > pH 8 > pH 8.5) (Bhowmick et al., 2009), while in *Bacillus subtilis* strain G7 and *Halomonas* sp. strain M-Cr, the opposite has been reported (i.e., pH 10 > pH 9 > pH 8 > pH 7 > pH 6) (Mary Mangaiyarkarasi et al., 2011; Mabrouk et al., 2014). The capability of strain AqSCr to reduce Cr(VI) under alkaline pH was further evaluated in LB medium (adjusted with the buffer Na_2_CO_3_⋅H_2_O) (Supplementary Figure 3). A one-way ANOVA analysis (Supplementary Table 4) of the reduction percentage showed statistically significant differences, with the Cr(VI) reduction higher under neutral than under alkaline conditions (pH 7 > pH 8 > pH 9) (Supplementary Figure 3 and Supplementary Table 4). In summary, the Cr(VI) resistance of strain AqSCr increases with increasing pH, while the reduction decreases. One possible explanation is that, it is the result of a higher rate of Cr(VI) transport under neutral pH than under alkaline conditions, as has been reported for the transport of sulfate. However, to our knowledge, no differences have been described yet in the transport of Cr(VI) at different pH by sulfate transporters [the main route of entry to the cell of Cr(VI)] (Pereira et al., 2008; Zolotarev et al., 2008; Zhang L. et al., 2014). Strain AqSCr is able to grow with anaerobic LB (data not shown); however, it was not possible to evaluate Cr(VI) reduction under these conditions due to high abiotic reduction in the controls.

The microbial ability to reduce Cr(VI) could be the result of enzyme-mediated oxidation–reduction reactions or by indirect physicochemical processes. Enzymatic reduction may or may not be associated with energy generation [Cr(VI) respiration]. In our study, NADPH-dependent chromate reductase activity was detected in the soluble protein fraction and not in the membrane fractions of cell extracts of *Klebsiella* sp. strain AqSCr (Figure 2C). We constructed a multiple mutant with all six genes encoding homologs of the soluble chromate reductases deleted (Supplementary Table 1). Interestingly, this mutant reduced Cr(VI) as well as wild type (Supplementary Figure 4), but the availability of NAD(P)H may constrain the reduction rate (see RNA-Seq results below).

### Metabolic Adaptation to Cr(VI)

**FIGURE 3:**
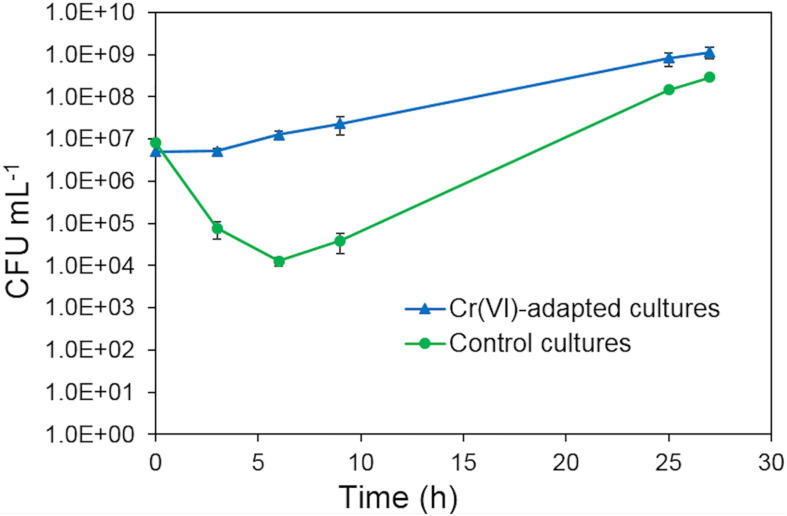
Growth of *Klebsiella* sp. strain AqSCr in LB medium (pH 8) with 11 mM Cr(VI), starting with cells with pre-exposure (blue triangles) and without pre-exposure (green circles).

The metabolic adaptation of strain AqSCr to Cr(VI) was tested aerobically in LB medium. To ensure that chromate ion was the predominant species, experiments were conducted at pH 8 with 11 mM Cr(VI) as a sublethal concentration; Figure 3. Cultures without Cr(VI) pretreatment showed a decrease in cell viability immediately after inoculation, and recovery of cell growth was observed after 9 h of incubation. Cultures preadapted with 11 mM Cr(VI) (two subculture steps) showed no decrease in cell viability and began to grow after 6 h of incubation. At 25 h, the preadapted cultures reached higher cell viability. This effect was similar to the one reported in *S. oneidensis* (Chourey et al., 2006) and suggested gene induction of Cr(VI) resistance mechanisms.

**FIGURE 4:**
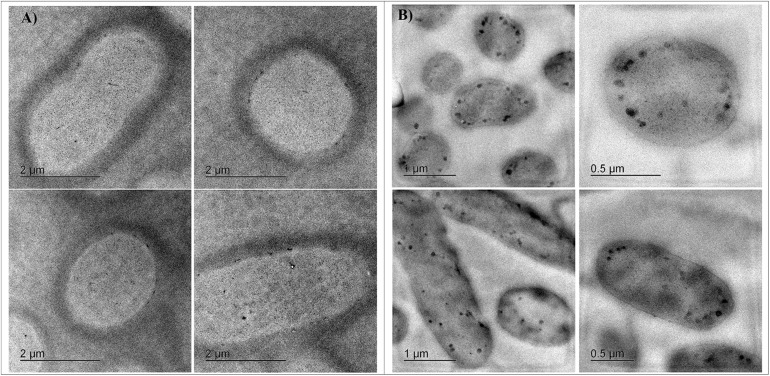
TEM Micrographs of *Klebsiella* sp. strain AqSCr cells grown in **(A)** LB (pH 8) without Cr(VI) and **(B)** LB (pH 8) with 11 mM Cr(VI).

Cells from cultures adapted to 11 mM Cr(VI) in LB at pH 8 were analyzed by transmission electron microscopy (TEM); micrographs showed dense spots distributed predominantly in the cellular internal periphery; however, these spots were absent in samples of cultures grown without Cr(VI) (Figures 4A,B), suggesting a localized chromate reduction.

### Transcriptional Profile of Cells of *Klebsiella* sp. Strain AqSCr Adapted to 11 mM Cr(VI)

The differential transcriptional profile of *Klebsiella* sp. strain AqSCr cells pre-adapted to 11 mM Cr(VI) relative to control in LB without Cr(VI) was evaluated by RNA-Seq. Three replicates of each condition were conducted from the mid-exponential growth phase for RNA extraction, library construction, and sequencing. Analysis of variation among RNA-Seq libraries showed differences between controls and Cr(VI)-adapted cells; a replicate of control treatment had a higher divergence from the other two control replicates but, nevertheless, was clearly distinct from the Cr(VI) treatment replicates (Supplementary Figure 5). Reads mapped over 5,097 genes; using a |log_2_FC| ≥ 1.5 and a *p*-adj ≤ 0.01 cutoff, 255 genes were upregulated, while 240 were downregulated in the presence of Cr(VI), respectively (Supplementary Figure 6). Genes associated with functional categories previously linked to metal resistance were manually curated (Supplementary Tables 5, 6). Only 20 genes harbored in plasmid DNA was differentially transcribed (Supplementary Table 7). To validate the RNA-Seq data, the transcription levels of 13 selected genes were further analyzed by RT-qPCR (see section “Materials and Methods” for details). Results showed a good correlation between RT-qPCR and RNA-Seq for the 13 selected genes (Table 1), with a Pearson correlation of 0.9615 (Supplementary Figure 7).

**TABLE 1:** Differential transcription of selected genes of strain AqSCr in the Cr(VI)-adapted state compared to control conditions, determined by two methods.

	RNA-Seq	qRT-PCR
	
ORF	Log_2_FC	2−ΔΔ Ct
*sulP* (BHE81_25425)	4.57	3.81
*rpoE* (BHE81_12570)	1.61	2.85
*gltA2* (BHE81_19460)	1.48	3.11
*cybB* (BHE81_07325)	1.88	14.88
*cybC* (BHE81_25140)	1.48	2.66
*recN* (BHE81_12780)	1.54	3.97
*lpdA* (BHE81_01240)	0.64	2.13
*pflB* (BHE81_12590)	−5.21	0.03
*cydB* (BHE81_19515)	−2.62	0.19
*gltA1* (BHE81_03385)	−4.35	0.03
*rpoB* (BHE81_23755)	0.012	1.21
*alaRS* (BHE81_13165)	−0.04	1.00
*sdaAB* (BHE81_09265)	−0.04	1.17

**FIGURE 5:**
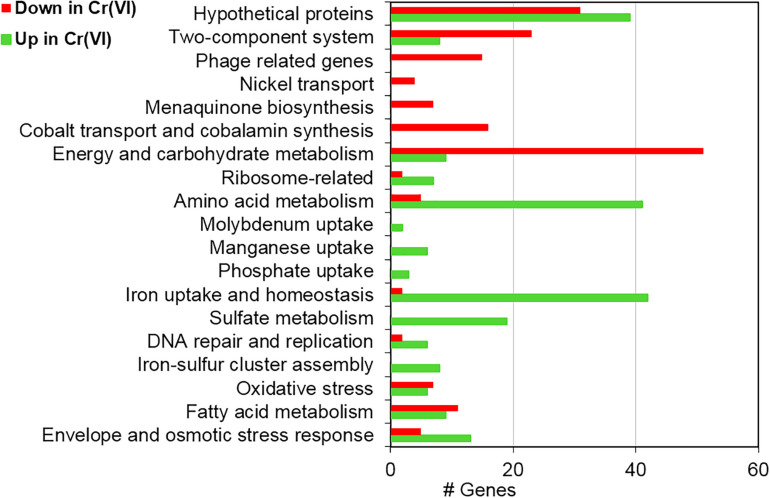
Functional assignment of differentially transcribed genes of strain AqSCr grown in the presence of 11 mM Cr(VI) in LB medium (pH 8) with respect to controls.

**FIGURE 6:**
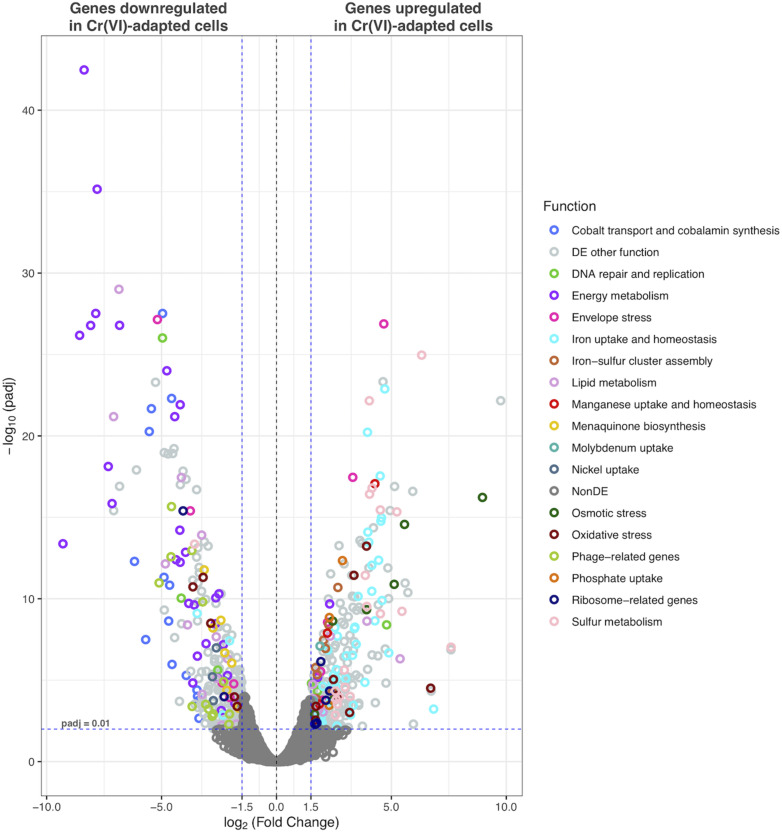
Volcano plot displaying differentially transcribed genes between Cr(VI)-adapted cells and controls. Each dot represents an ORF. ORFs with *p*-adj ≤ 0.01 and |Log2FC| > 1.5 were considered as differentially transcribed. Genes with putative functions previously associated with Cr(VI)-response in other bacteria are highlighted.

**FIGURE 7:**
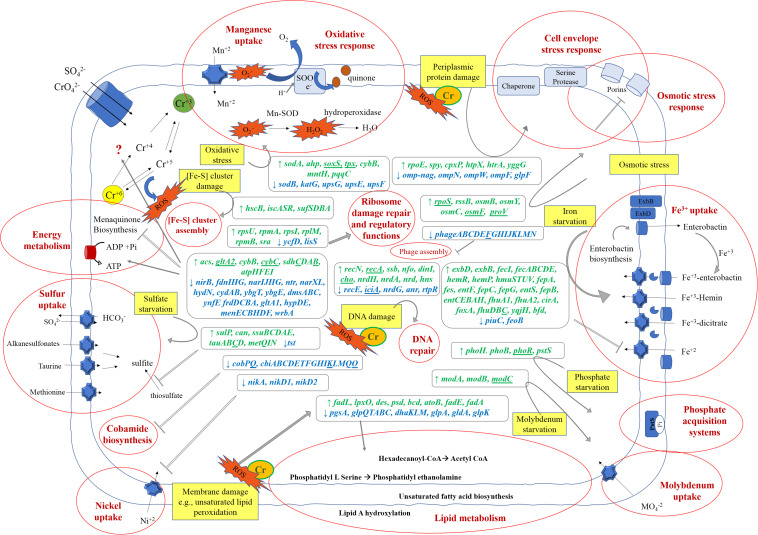
Schematic model of *Klebsiella* sp. strain AqSCr metabolic adaptation to 11 mM Cr(VI). Stress pathways elicited by Cr(VI) (yellow squares) and metabolic responses (written in red bold inside red circles) associated to the upregulated (green) and downregulated genes (blue) in the presence of Cr(VI) are highlighted. Gene abbreviations correspond to those in Supplementary Tables 5, 6. Genes underlines are those that were found to be differentially transcribed but outside of the cutoff parameters.

Cr(VI) promotes significant changes in the global gene transcription of *Klebsiella* sp. strain AqSCr (Figures 5, 6). The Cr(VI)-adapted state of strain AqSCr involves mostly the differential transcription of genes related to oxidative stress response, DNA repair and replication response, sulfur, iron, phosphate, and molybdenum acquisition systems, osmotic-envelope stress response, lipid metabolism, energy metabolism, amino acid metabolism, ribosomal subunits, cobalamin synthesis, and bacteriophage assembly (Figure 7 and Supplementary Tables 5, 6). It is noteworthy that none of the eight genes encoding homologs of Cr(VI) reductases (soluble or membrane-bound) nor the *chrA* and *chrB* genes was differentially transcribed in the Cr(IV)-adapted culture vs. the control (Supplementary Table 1). These results are consistent with results obtained with the mutants described in this study, indicating that reduction and resistance of Cr(VI) in strain AqSCr is independent of these systems.

#### Oxidative Stress Response

The best studied mechanism of chromium toxicity is related to oxidative stress. It involves an unspecific oxidation cascade initiated by ROS and H_2_O_2_ (Shi and Dalal, 1990; Jones et al., 1991; Branco and Morais, 2016; Wakeel et al., 2020). In strain AqSCr, genes encoding a Mn-superoxide dismutase (SodA) (BHE81_23030), two alkyl hydroperoxidases (BHE81_13040, BHE81_06815), a lipid hydroperoxide peroxidase (BHE81_04165), and the cytochrome CybB (BHE81_07325), recently described as a superoxide oxidase (SOO) in *E. coli*, were upregulated in the presence of Cr(VI) (Supplementary Table 5). These proteins are potentially able to destroy both superoxide (O_2_^–^) and peroxide (H_2_O_2_) (Branco and Morais, 2016; Lundgren et al., 2018). In addition, gene BHE81_06560 that encodes a pyrroloquinoline-quinone synthase PqqC, which is the last of six proteins involved in the synthesis of pyrroloquinoline quinone (PQQ), was also upregulated (Supplementary Table 5). This is a redox cofactor from several prokaryotic and eukaryotic enzymes that can act as a ROS scavenger (Magnusson et al., 2004; Kumar and Kar, 2015). The genes encoding the other five proteins involved in PQQ synthesis (BHE81_06550, BHE81_06555, BHE81_06560, BHE81_06565, and BHE81_06570) were also upregulated in the Cr(VI)-adapted culture vs the control but below the cutoff level.

The gene encoding the transcriptional regulator SoxS (BHE81_24245) was upregulated with a log_2_FC = 1.74, but with a *p*-adj of 0.012 slightly above of the cutoff level (≤0.01); however, it could influence Cr(VI) adaptation since its role in oxidative stress has been observed in several microorganisms (Seo et al., 2015). Furthermore, under oxidative stress, thiol groups are particularly vulnerable (Ayala-Castro et al., 2008). In strain AqSCr, the genes encoding the ISC (BHE81_12370, BHE81_12370 BHE81_12375, BHE81_12385, and BHE81_12390) and SUF systems (BHE81_08295, BHE81_0300, BHE81_08310, and BHE81_08315) involved in iron–sulfur cluster biogenesis and previously associated with oxidative stress response (Aguirre et al., 2013) were also upregulated in the Cr(VI)-adapted cultures vs the controls (Supplementary Table 5). In contrast, genes encoding a catalase/peroxidase enzyme (BHE81_06605), a Fe-superoxide dismutase (SodB, BHE81_07530) and the genes *upsE* (BHE81_04980), *upsF* (BHE81_04460), and *upsG* (BHE81_19080) were downregulated (Supplementary Table 6).

Superoxide dismutase enzymes (SOD) are primary elements in oxidative stress response. It has been proposed that Fe-SOD responds to general stress while Mn-SOD is induced by specific stimuli (Aguirre et al., 2013). It has also been proposed that Mn-SOD evolved from Fe-SOD in environments with low iron concentrations (Aguirre et al., 2013). In strain AqSCr, *sodA*, encoding a Mn-SOD, and *sodB*, encoding a Fe-SOD, genes were up- and downregulated in the presence of Cr(VI), respectively, probably due to the iron limitation induced by Cr(VI) (Supplementary Tables 5, 6). In *Ochrobactrum tritici* 5bvl1, *chrC*, encoding a Fe-SOD, appears to have a greater contribution than *chrF*, encoding a Mn-SOD, in the elimination of ROS during exposure to Cr(VI) (Branco and Morais, 2016). In *S. oneidensis*, a Fe-SOD was previously found to be upregulated in response to Cr(VI) (Brown et al., 2006; Chourey et al., 2006), while in *Caulobacter crescentus*, a Mn-SOD was upregulated in response to chromium, cadmium, and uranium based on gene transcription data by using microarrays (Hu et al., 2005). In *Staphylococcus aureus LZ-01*, according to the RNA-Seq data, superoxide dismutase-encoding genes were not differentially transcribed in response to Cr(VI) (Zhang X. et al., 2014). Overall, these reports suggest that Mn-SOD and Fe-SOD have different contributions to Cr(VI) resistance in different microorganisms.

Furthermore, unsaturated membrane lipids are major targets of oxidative stress; they are attacked by free radicals, and their degradation products, as malonaldehyde, can diffuse and act as secondary toxic messengers (Cabiscol et al., 2000). Alkyl hydroperoxidases act over alkyl peroxides rendering water and hydroxylated FAs alleviating oxidative stress (Hillas et al., 2000). The role of alkyl hydroperoxidases in chromate resistance has not been studied; thus, *ahp* genes (BHE81_13040 and BHE81_06815) upregulated in response to Cr(VI) would be attractive for future studies.

#### DNA Repair and Replication

Chromium elicits DNA damage both directly and by inducing oxidative stress. In Cr(VI)-adapted cells of strain AqSCr, genes encoding RecN (BHE81_12780), HNS (BHE81_08655), Nfo (BHE81_10860), Ssb (BHE81_26455), NrdH (BHE81_13045), NrdA (BHE81_13055), NrdB (BHE81_13060), and DinI (BHE81_21170 and BHE81_21980) were upregulated with respect to the controls (Supplementary Table 5). RecA (BHE81_13175) was also upregulated but below the cutoff level, with a log_2_FC = 1.23 (Supplementary Table 5). RecA and RecN are involved in recombinational repair of DNA double-strand breaks (Uranga et al., 2017). The gene *cho* (BHE81_03730), encoding a putative deoxyribonuclease, was also upregulated but with a *p*-adj slightly greater than (0.0106) the cutoff level (Supplementary Table 5). Moreover, RecE (BHE81_21560), NrdG (BHE81_25210), and RtprB (BHE81_25215), also involved in DNA repair and replication, were downregulated with respect to the controls (Supplementary Table 6).

#### Sulfur, Phosphate, Molybdate, Iron, and Manganese Uptake

Sulfate starvation induced by chromate stress has been reported in diverse microorganisms as a consequence of chemical homology between these oxyanions, which causes sulfate import inhibition (Aguilar-Barajas et al., 2011). To cope with sulfate limitation, microorganisms use diverse mechanisms including the expression of highly specific sulfate transporters, or utilization of alternative pathways to acquire sulfur.

In strain AqSCr, the gene *sulP* (BHE81_25425), encoding a putative sulfate permease from the MFS superfamily, was upregulated (log_2_FC = 4.57) with respect to the controls; the adjacent gene, *can* (BHE81_25430), encoding a putative carbonic anhydrase, was also upregulated (log_2_FC = 6.43), suggesting *sulP* encodes a sulfate/bicarbonate antiporter (Supplementary Table 5). BHE81_11720 gene, encoding a protein with 82.61% identity to the high-affinity, high-specificity sulfate transporter CysZ from *E. coli* (Zhang X. et al., 2014), was also upregulated (log_2_FC = 1.6) but with a *p*-adj (0.065) that was above the cutoff level. The gene cluster *cysPTWA* and the other two *sulP* genes were not differentially transcribed. Moreover, the gene BHE81_02200, encoding an arylsulfatase, which has been associated with high-affinity sulfate transport systems by liberating sulfate anion from esterified organic sulfate (Kertesz et al., 1993; Davies et al., 1999), and is induced under sulfate limitation, was found upregulated (log_2_FC = 2.78) (Supplementary Table 5). Additionally, three alternative pathways of sulfur acquisition: the taurine transport system (*tauABCDE*; BHE81_02450, BHE81_02455 BHE81_02460, and BHE81_02465), the alkanesulfonates transport system (*ssuEADCB;* BHE81_20690, BHE81_20685, BHE81_20680, BHE81_20675, and BHE81_20670), and the methionine transport system (*metQLN*; BHE81_04410, BHE81_04415, and BHE81_04420), were upregulated (Supplementary Table 5). The *tst* gene (BHE81_03655), encoding a putative thiosulfate sulfur transferase, was downregulated (log_2_FC = −3.57) (Supplementary Table 6).

Phosphate transport inhibition by chromate due to chemical homology has been reported previously (Aguilar-Barajas et al., 2011). In strain AqSCr, Cr(VI)-adapted cells, *phoH* gene (BHE81_21040; log_2_FC = 2.32), and the phosphate regulon two-component system genes *phoB* (BHE81_02595; log_2_FC = 2.33) and *phoR* (BHE81_02600; log_2_FC = 1.33; below of our cutoff level) (Supplementary Table 5) were upregulated. In addition, the gene *pstS* (BHE81_2262) encoding a periplasmic phosphate-binding protein of a phosphate ABC transporter was also upregulated with a log_2_FC = 2.9; however, the genes encoding the permease and the ATP-binding protein of this transporter (BHE81_22610, BHE81_22615, and BHE81_22620) were not. The upregulation of these genes suggests a phosphate starvation response.

Molybdenum (Mo)-containing enzymes participate in the metabolism of nitrogen, sulfur, and carbon compounds; their biosynthesis involves an intricate process in which molybdate uptake is essential. Molybdate uptake could be performed by sulfate transporters. Only under conditions of limitation is a high-affinity molybdate transport system, ModABC, consisting of a periplasmic molybdenum-binding protein ModA, a permease ModB, and an ATP-binding protein ModC, induced (Aguilar-Barajas et al., 2011). In strain AqSCr, the genes *modA* (BHE81_19645, log_2_FC = 1.63), *modB* (BHE81_19650, log_2_FC = 1.92), and *modC* (BHE81_19655, log_2_FC = 1.24; below the cutoff level) were upregulated in Cr(VI)-adapted cells with respect to the controls, probably due to molybdate transport inhibition by Cr(VI) through sulfate transporters. ModA of *Escherichia coli* has high affinity to Cr(VI) although its role in Cr(VI) resistance has not been yet demonstrated; therefore, it could potentially recruit/bind Cr(VI) in the periplasm, diminishing DNA and other cytoplasmic damage. It is still unknown if ModB and ModC can transport chromate through the inner membrane (Karpus et al., 2017). However, Gang et al. (2019) reported that levels of ModA and ModC proteins of *S. oneidensis* MR-1 increased during long-term exposure to Cr(VI), pointing out its role in Cr(VI) response.

In diverse microorganisms, Cr(VI) exposure leads to iron starvation and, in addition, it has been suggested that Cr(III) interferes with cellular iron uptake; however, the mechanisms remain unclear (Wang and Newton, 1969; Hu et al., 2005; Thompson et al., 2007, 2010; Viti et al., 2014). In *Klebsiella* sp. strain AqSCr, 47 genes related to iron uptake were upregulated in the presence of Cr(VI) (Supplementary Table 5), most of them are organized in clusters. These genes encode four different systems of Fe(III) uptake: (1) FecABCDE, a Fe(III)-citrate transporter; (2) HemR, a TonB-dependent hemin ferrichrome receptor and HmuSTUV, a hemin ABC transporter; (3) EntCEBAH and EntF, involved in enterobactin biosynthesis, EntS, a MFS enterobactin exporter, FepA, a TonB-dependent ferric enterobactin receptor, and FepBCG, a Fe(III)-enterobactin ABC transporter; and (4) FhuDBC, a Fe(III) ABC transporter. Also upregulated were *fecI* (BHE81_26615; log_2_FC = 3.06), encoding a sigma factor; *exbB* (BHE81_15090; log_2_FC = 2.21), and *exbD* (BHE81_15085; log_2_FC = 2.43), encoding the TonB-dependent energy transduction system; *yqjH* (BHE81_15690; log_2_FC = 2.04), encoding a NADPH-dependent ferric siderophore reductase; and *fbd* (BHE81_16810; log_2_FC = 5.3), encoding a Bacterioferritin-associated ferredoxin. In contrast, BHE81_03845 and BHE81_17105, encoding the iron-uptake factor PiuC (log_2_FC = −3.49), and a putative ferrous iron permease FeoB (log_2_FC = −2.03), were downregulated (Supplementary Table 6). The upregulation of genes related to sulfur and iron uptake could probably be partly due to an increased demand for protective thiol and Fe-S-containing compounds needed for coping with the oxidative stress imposed by Cr(VI).

Furthermore, manganese (Mn) is an essential trace element and plays a key role in oxidative stress adaptation, as a cofactor of superoxide dismutase, by protective replacement of Fe^2+^ as a cofactor, and by formation of non-enzymatic complexes that destroy superoxide (Inaoka et al., 1999; Culotta and Daly, 2013). In strain AqSCr, two manganese transporter systems, *sitABCD* and *mntH*, were found to be upregulated with respect to the control, further supporting the role of Mn in the coping mechanism of high Cr(VI) concentrations (Supplementary Table 5).

#### Cell Envelope and Osmotic Stress Response

We found the gene encoding the alternative sigma factor RpoE (BHE81_12570) upregulated with a log_2_FC = 1.61 (Supplementary Table 5). This sigma factor has been reported to mediate the bacterial envelope stress response, activated by the accumulation of misfolded and/or mistranslocated outer-membrane proteins or lipopolysaccharides within the periplasm (Rowley et al., 2006; Hews et al., 2019). RpoE has been implicated in response to diverse types of stress that compromise cell envelope integrity, including heat shock (Rouvière et al., 1995), oxidative stress (Dai et al., 2015), and osmotic stress (Bianchi and Baneyx, 1999). Also, it has been reported that it is implicated in resistance to Zn(II), Cd(II), and Cu(II) in *E. coli* (Egler et al., 2005), and to Zn(II), Ni(II), Co(II), and Cu(II) in *Cupriavidus metallidurans* (Grosse et al., 2007). However, its specific association to Cr(VI) resistance has not previously been reported. In relation to the envelope stress response, the genes *spy* (BHE81_03725) and *cpxP* (BHE81_23050), encoding periplasmic chaperones, and *htpX* (BHE81_09355), *htrA* (BHE81_01550), and *yggG* (BHE81_14840), encoding a heat shock membrane protease (Huang et al., 2008; Hews et al., 2019), were also upregulated (Supplementary Table 5), while the genes *ompN* (BHE81_10030), *ompW* (BHE81_03840), and *ompF* (BHE81_20620), encoding outer membrane proteins, were downregulated (Supplementary Table 6), consistent with the RpoE regulon activation (Hews et al., 2019).

BHE81_04010, BHE81_00470, and BHE81_27505 genes, encoding the outer membrane protein OsmB, the outer membrane-bound periplasmic chaperone OsmY, and the internal membrane protein OsmC, respectively, were also upregulated in the presence of Cr(VI) (Supplementary Table 5). These genes have previously been associated with osmotic stress response (Jung et al., 1990). OsmC of *E. coli* has also been reported to be involved in H_2_O_2_ resistance (Conter et al., 2001). BHE81_13545, which encodes the sigma factor RpoS related to osmotic stress-stationary phase, was slightly upregulated below the defined cutoff with a log_2_FC = 0.99 (Supplementary Table 5). In *S. typhi*, Du et al. (2011) reported that 38 genes (including *osmC*, *osmY*, and *osmB*) were co-regulated by sigma E and sigma S during hyperosmotic stress.

Additionally, a primary consequence of unsaturated lipid damage by peroxidation is a decrease in membrane fluidity, disrupting several cellular functions (Cabiscol et al., 2000). This could be alleviated by the production of new unsaturated FAs (Zhang et al., 2015). In strain AqSCr, significant differences were found in genes involved in the synthesis, modification, and degradation of membrane-forming components. In Cr(VI)-adapted cells, two genes encoding alkyl peroxidases (BHE81_13040 and BHE81_06815) and a lipid hydroperoxide peroxidase (BHE81_04165) were found to be upregulated (above-mentioned), probably in response to lipid peroxidation. In addition, the *des* gene (BHE81_09095), encoding a putative FA desaturase, was also upregulated with a log_2_FC = 5.41. The *fabA* gene (BHE81_20740), encoding the putative 3-hydroxyacyl-[acyl-carrier-protein] dehydratase, which is involved in *de novo* biosynthesis of unsaturated FAs, was slightly upregulated with a log_2_FC = 0.89 (FC = 1.85), suggesting the activation of a common mechanism of desaturation of membrane FAs (Aguilar and Cronan, 1998; Aguilar and De Mendoza, 2006), probably as a mechanism to allow replenishing unsaturated lipids by the novo synthesis as has been reported for *Pichia pastoris* (Zhang et al., 2015). Moreover, the genes *bcd* (BHE81_08150), *atoB* (BHE81_14340), *fadE* (BHE81_01820), and *fadA* (BHE81_23670), encoding proteins involved in FA degradation, were upregulated with log_2_FC values of 4.0, 2.0, 2.6, and 2.37, respectively, probably for the degradation of unsaturated FAs damaged.

In addition, genes *lpxO* (BHE81_13000) and *psd* (BHE81_24760), encoding a putative dioxygenase involved in lipid A hydroxylation and a putative phosphatidylserine decarboxylase involved in phosphatidylethanolamine biosynthesis, respectively, were upregulated in Cr(VI)-adapted cells (Supplementary Table 5), while *pgsA* (BHE81_03650), *glpQ* (BHE81_11130), and *dhaKLM* (BHE81_15670, BHE81_15675, and BHE81_15680) were downregulated with respect to the controls (Supplementary Table 6).

#### Ribosomal Functions

There are some reports of increased synthesis of specific ribosomal subunits in response to stress conditions to compensate for the low rate of translation initiation (O’Connell and Thomashow, 2000). Recently, this effect was reported for Cr(VI) stress in *Pseudomonas aeruginosa* (Kılıç et al., 2010) and *S. oneidensis* (Bencheikh-Latmani et al., 2005; Chourey et al., 2006). Here, we found that five genes encoding ribosomal subunits S9 (BHE81_16380), S21 (BHE81_15345), S22 (BHE81_15345), L13 (BHE81_16385), L27 (BHE81_16205), and L28 (BHE81_18145) were upregulated (Supplementary Table 5), while *ycfD* (BHE81_25675), encoding a putative hydroxylase of the 50S ribosomal protein L16, was downregulated (Supplementary Table 6) with respect to the controls, supporting the idea that Cr(VI) induce ribosome modifications.

#### Energy Production

The Cr(VI)-adapted state of strain AqSCr involves the differential transcription of genes involved in energy metabolism. We found that the gene *gltA2* (BHE81_19460), encoding a citrate synthase, was upregulated with a log_2_FC = 1.48 (slightly below cutoff level) (Supplementary Table 5), while its ortholog *gltA1* (BHE81_03385) was downregulated with a log_2_FC = −4.35 (Supplementary Table 6). The differential expression of these genes was confirmed by RT-qPCR (Table 1). *gltA* orthologs are present in *Klebsiella* spp.; however, their preferential use under stress conditions, to our knowledge, has not been reported in any organisms. Also, the genes encoding the succinate dehydrogenase complex (BHE81_19465, BHE81_19470, and BHE81_19475), the membrane cytochrome *b*_561_ (BHE81_07325), the soluble cytochrome *b*_562_ (BHE81_25140), and four ATP synthase subunits (BHE81_22675, BHE81_22680, BHE81_22685, and BHE81_22695), were upregulated (Supplementary Table 5). It is not clear whether the upregulation of ATP synthase subunits is due to high sensitivity to Cr(VI) (or oxidative stress) or to a higher energy demand.

Genes encoding the fumarate reductase complex (BHE81_24725, BHE81_24730, BHE81_24735, and BHE81_24740), the cytochrome *bd* oxidase complex (BHE81_19510 and BHE81_19515), and several genes involved in nitrate reduction including the NarXL two-component system (BHE81_08735 and BHE81_08740), a nitrate/nitrite transporter MFC type (BHE81_08730), the nitrate reductase complex (BHE81_08705, BHE81_08710, BHE81_08715, and BHE81_08720), the formate dehydrogenase N complex (nitrate-dependent) (BHE81_06840, BHE81_06845, and BHE81_06850), as well as several genes involved in menaquinone biosynthesis were downregulated (Supplementary Table 6), suggesting Cr(VI)-induced changes in the electron flow in the respiratory chain.

#### Other Genes Differentially Transcribed

In addition to the differentially transcribed genes described above, we found *cobPQ* (BHE81_13895 and BHE81_13900) and *cbiABCDETFGHIKLMQO* gene cluster involved in cobalamin biosynthesis, *nikA* (BHE81_17475), *nikD1* (BHE81_17495), and *nikD2* (BHE81_17500) related to nickel uptake, and several genes related to phage assembly downregulated (Supplementary Table 6), in contrast with the report of Chourey et al. (2006) in which prophage-related genes of *Shewanella* were upregulated in response to Cr(VI).

Supplementary Table 8 includes the 50 most highly upregulated genes of strain AqSCr in the Cr(VI) adapted state, some of them were mentioned above, a FA desaturase is also present, as well as some genes involved in iron uptake. Furthermore, genes whose role in chromate resistance or reduction has not previously been reported or discussed are also present.

Finally, this work shows a total of 39 upregulated genes and 31 downregulated genes encoding hypothetical proteins whose function could reveal novel mechanisms involved in bacterial resistance to Cr(VI).

Only a few studies of bacterial global response to Cr(VI) have been reported to date; these include proteomic and transcriptional studies; some of them correspond to short-term exposure to Cr(VI) (initial stress response) and the others correspond to long-term exposure (an adapted state) (Bencheikh-Latmani et al., 2005; Hu et al., 2005; Brown et al., 2006; Chourey et al., 2006, 2008; Thompson et al., 2007, 2010; Henne et al., 2009; Monsieurs et al., 2011; Zhang X. et al., 2014; Zhao et al., 2014; Chai et al., 2019; Gang et al., 2019; Shah and Damare, 2019). Transcriptional response can be assessed by microarrays or by RNA-Seq; however, the latter method is superior in detecting low abundance transcripts, differentiating isoforms, and has a broader dynamic range, allowing the detection of more differentially transcribed genes with higher fold change (Zhao et al., 2014). To date, only one transcriptional study has been conducted by RNA-Seq analysis; it corresponds to a short-term exposure to Cr(VI) (Zhang X. et al., 2014). Figure 7 shows a schematic representation of the cellular mechanisms activated in the Cr(VI)-adapted state of strain AqSCr, and a summary of differentially transcribed genes is presented. To our knowledge, this is the first report of transcriptional profiling of bacterial long-term exposure to Cr(VI) performed by RNA-Seq, which could be useful to improve the bacterial characteristics or culture conditions for its application in waste treatment.

### Fatty Acid Unsaturation and Hydroxylation Profile in Cr(VI)-Adapted Cells

**FIGURE 8:**
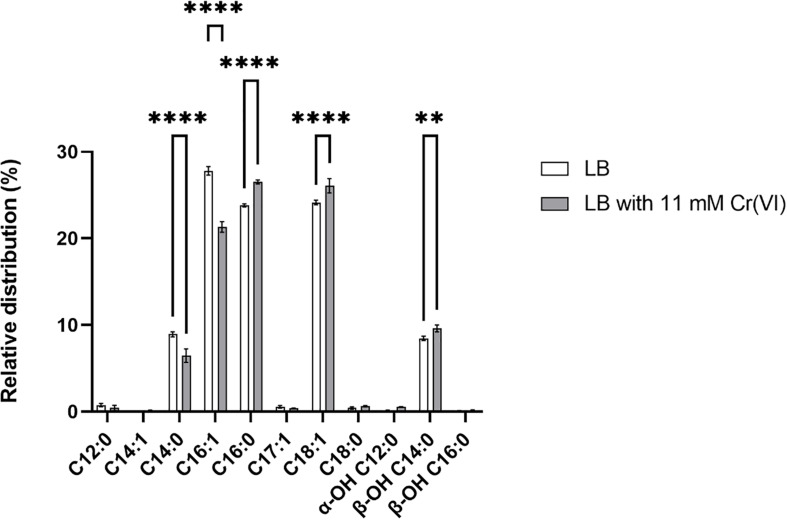
Relative distribution of fatty acid methyl esters (FAMEs) of *Klebsiella* sp. strain AqSCr cells adapted to 11 mM Cr(VI) or grown in LB (pH 8) (controls). Each column represents the mean ± SEM of three independent replicates. Significant difference for specific FAMEs compounds of Cr(VI)-treated cells vs. control (LB), using a two-way ANOVA followed by a Sidak’s multiple comparison test. ****p < 0.0001, **p < 0.0016, *n* = 3. Bars represent mean ± SEM.

Membranes are the first protection of cells to external factors, and their modification in response to a variety of stresses has been reported. However, the modifications under chromium stress have rarely been studied, primarily by TEM approaches (Francisco et al., 2010; Fernández et al., 2017). Here, transcriptomic studies showed differential transcription of genes involved in the synthesis, modification, and degradation of membrane components, including the upregulation of a FA desaturase, a 3-hydroxyacyl-[acyl-carrier-protein] dehydratase, and two alkyl peroxidases. We investigated the composition of FAs (Figure 8 and Supplementary Table 9) of strain AqSCr grown in LB medium (control condition) and Cr(VI)-adapted cells.

The membrane lipid analysis performed in our study showed that the cells of strain AqSCr contained the C_14:1_, C_16:1_, C_17:1_, and C_18:1_ unsaturated FAs and the C_12:0_, C_14:0_, C_16:0_, and C_18:0_ saturated FA species. The relative distribution of the unsaturated C_16:1_ FAs decreased in Cr(VI)-adapted cells with respect to the controls, accounting for 27.8% in the control condition compared to 21.3% in the Cr(VI)-adapted cells. A minor increase (1.9%) of the C_18:1_ FA was found in those Cr(VI)-adapted cells when compared to the control (Figure 8 and Supplementary Table 9). The hydroxyl FA composition was slightly increased in Cr(VI)-adapted cells. Specifically, the α-OH C_12:0_ increased from 0.1 to 0.6%, and the β-OH C_14:0_ increased from 8.5 to 9.6%, while no changes were detected in the β-OH C_16:0_ FA compared to the β-OH C_16:0_ FA distribution of cells grown in LB. The slightly increased content of hydroxyl FAs in Cr(VI)-adapted cells could result in the action of the LpxO dioxygenase on the FA of Lipid A, whose gene was found upregulated in our study. These changes could represent membrane adaptation (Vences-Guzmán et al., 2012) or the result of a repair process. We hypothesize that, in the presence of Cr(VI), the peroxidation of unsaturated lipids conduces to their degradation, therefore requiring compensation by a continuous *de novo* synthesis of unsaturated FAs, which is probably the reason for the FA desaturase gene upregulation.

## Conclusion

*Klebsiella* sp. strain AqSCr isolated and characterized in this work reduces Cr(VI) both aerobically and anaerobically and is highly resistant to Cr(VI) up to 34 mM; surprisingly, this resistance is independent of the ChrA efflux transporter. The metabolic response to Cr(VI) involves the differential transcription of 495 genes involved in oxidative stress response, DNA repair and replication, sulfur uptake, iron uptake, envelope-osmotic stress response, ribosomal functions, and energy metabolism. From 255 upregulated genes, 47 and 20 were involved in iron and sulfur uptake, respectively, suggesting their prominent role in chromium resistance.

Moreover, three genes encoding alternative sigma factors, *fecl*, *rpoE*, and *rpoS*, were upregulated, indicating their role in orchestrating Cr(VI) resistance mechanisms. Summing up, here we report the upregulation of genes not previously associated with chromium resistance, as *cybB*, encoding a putative SOO, *gltA2*, encoding an alternative citrate synthase, and *des*, encoding a FA desaturase. We also found a lower proportion of the C_16:1_ unsaturated FA in the membrane of Cr(VI)-adapted cells with respect to controls, which suggests that the upregulation of a FA desaturase and a 3-hydroxyacyl-[acyl-carrier-protein] dehydratase are involved in compensating the unsaturated lipid peroxidation and degradation, by *de novo* synthesis. This study could help to manipulate and improve bacterial culture conditions for their application in the treatment of Cr(VI)-contaminated waste.

## Data Availability Statement

The datasets generated for this study can be found in the GenBank repository (https://www.ncbi.nlm.nih.gov/nuccore) with accession number SAMN05730075 and in NCBI’s Gene expression Omnibus repository (https://www.ncbi.nlm.nih.gov/geo) accessible through GEO series accession number GSE160968.

## Author Contributions

PL contributed to the conceptualization, investigation, and formal analysis. KJ designed, supervised, and coordinated the study. LV-A performed the RNA-Seq statistical analyses. FR-M and EM performed the genome assembling. DS-C, MK, and LV performed lipid analysis and interpretation of the results. PL, DS-C, EM, LV, and KJ wrote and edited the manuscript. All authors contributed to the article and approved the submitted version.

## Conflict of Interest

The authors declare that the research was conducted in the absence of any commercial or financial relationships that could be construed as a potential conflict of interest. The reviewer HR-R declared a shared affiliation, with no collaboration, with one of the authors, LV-A, to the handling editor at the time of the review.

## Footnotes

1 http://www.uusmb.unam.mx/ideamex/

2 http://www.kegg.jp/ghostkoala/

3 https://www.graphpad.com
