# Supplementary material for: Transcriptome Analysis Reveals Cr(VI) Adaptation Mechanisms in Klebsiella sp. Strain AqSCr
Source: Front Microbiol. 2021 May 27;12:656589. doi: 10.3389/fmicb.2021.656589 (PMC8195247; doi:10.3389/fmicb.2021.656589)
Supplement: Supplementary file 1 [file Data_Sheet_1.PDF]

Supplementary Material

1 Supplementary Figures and Tables

1.1 Supplementary Figures

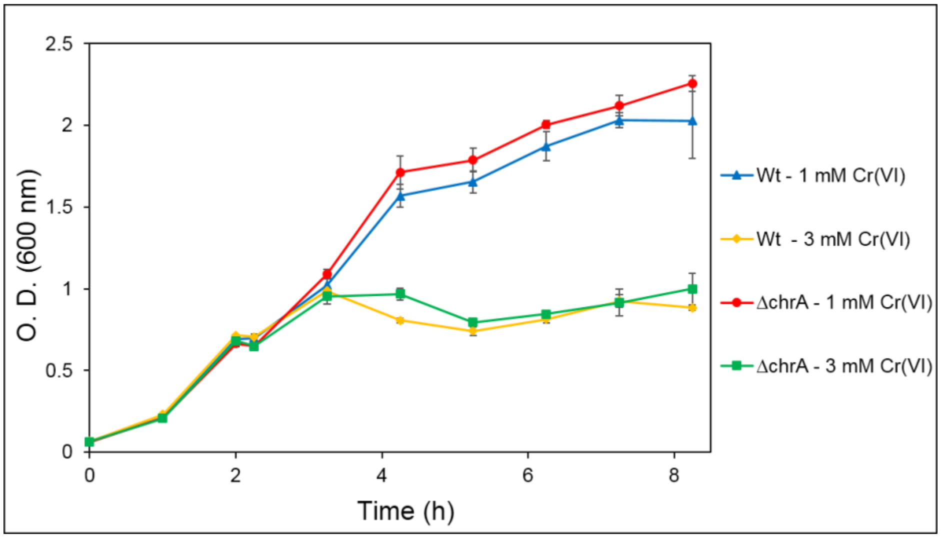

Figure S1. Growth of strain AqSCr wild type and the Δ*chrA* mutant in LB broth medium with 1 mM or 3 mM Cr(VI). Data are presented as mean +/- standard error of the mean.

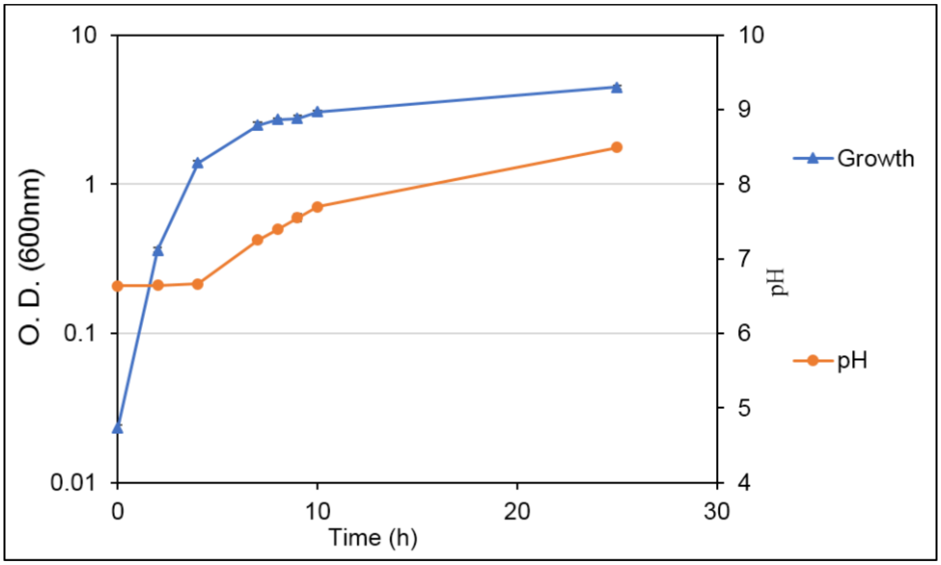

Figure S2. Aerobic growth of *Klebsiella* sp. strain AqSCr in unbuffered LB medium and pH changes. Data are presented as mean +/- standard error of the mean.

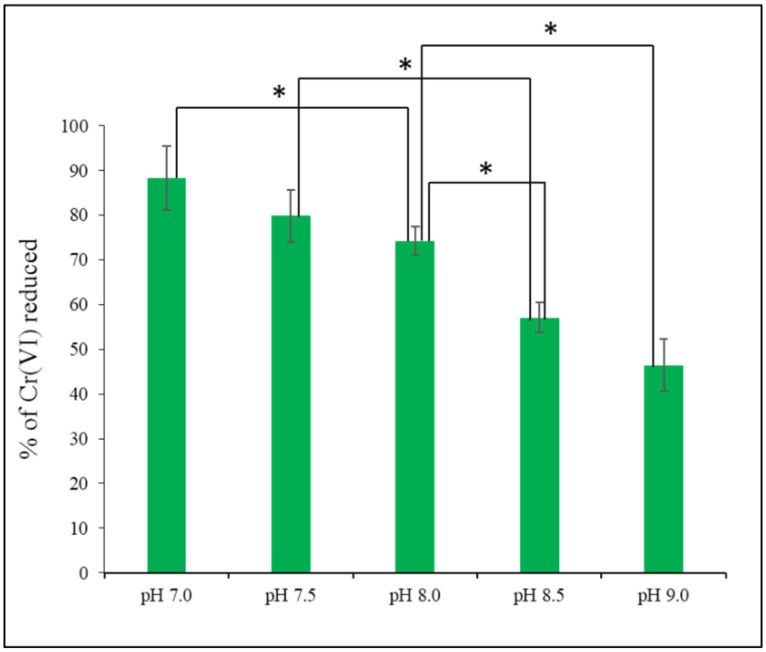

Figure S3. Reduction of Cr(VI) 100 µM after 24 hours by *Klebsiella* sp. strain AqSCr in aerobic LB medium at different pH values. Each column represents the mean of three independent replicates. Bars represent mean +/- standard error of the mean. Significant differences in reduction of Cr(VI) were determined by one-way ANOVA test (Table S4) . *$p < 0.05$.

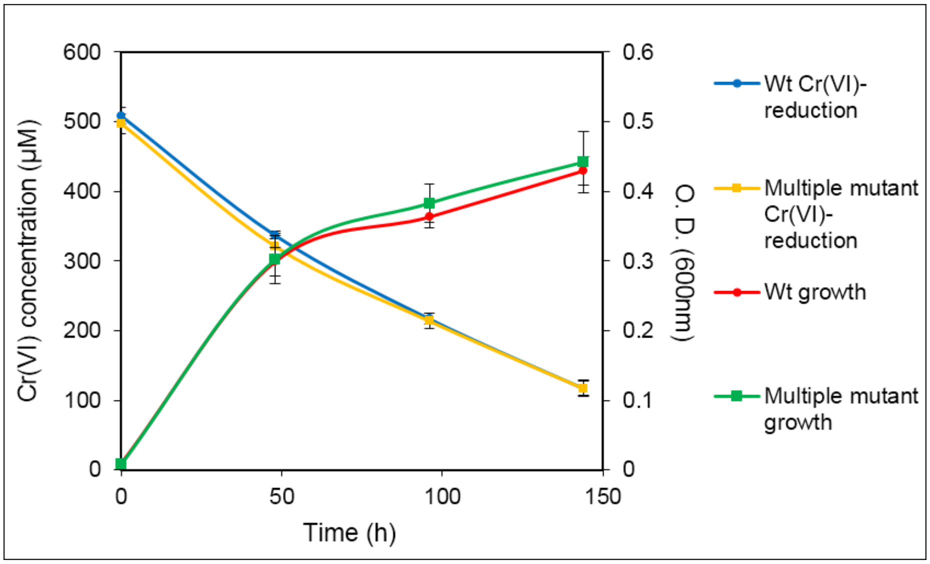

Figure S4. Growth and Cr(VI) reduction in LB (pH 7) containing Cr(VI) 500 µM by strain AqSCr wild type and the multiple mutant (six putative soluble chromate reductases were deleted – see text). Data are presented as mean +/- standard error of the mean.

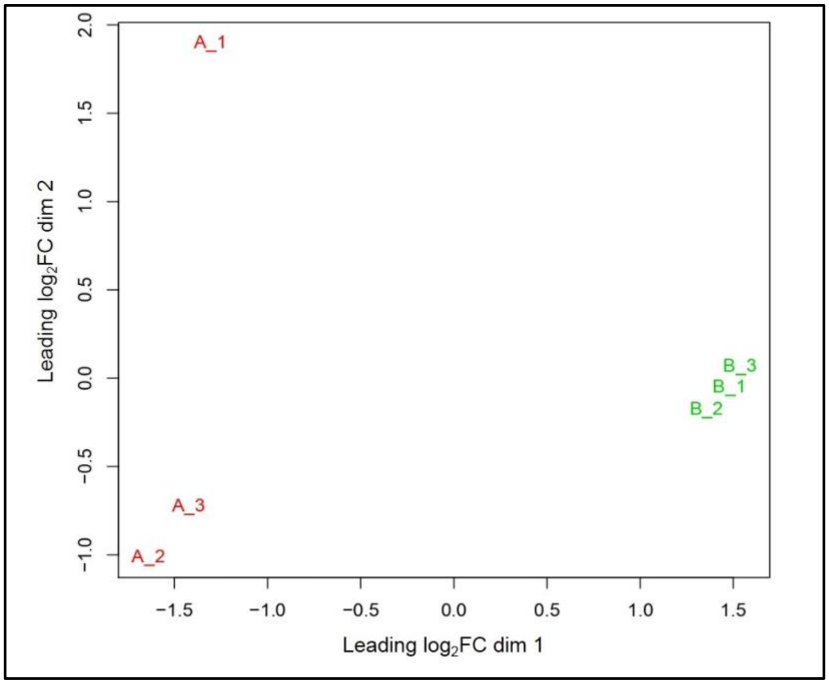

Figure S5. Multidimensional scaling (MDS) plot of RNA-Seq data. A) controls and B) 11 mM Cr(VI)-adapted cells.

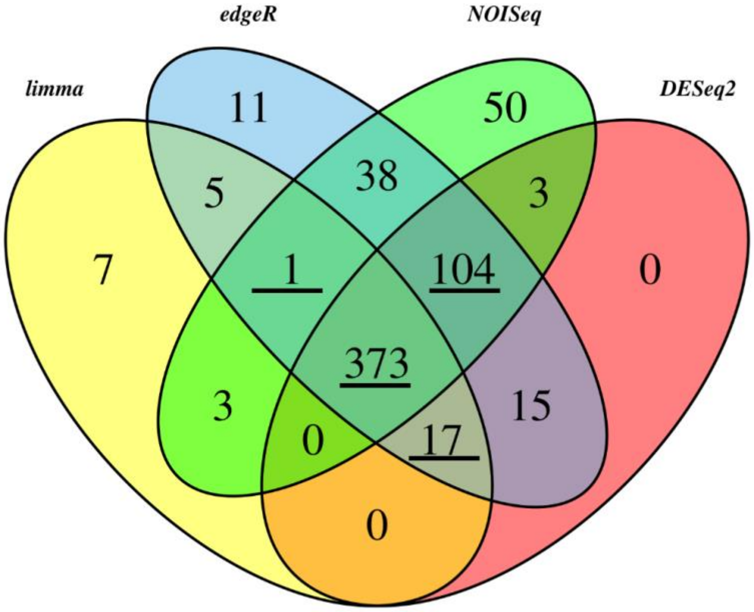

Figure S6. Venn diagram of RNA-Seq data processing. Depicting the number of ORFs differentially transcribed (genes with at least ten counts per million, FDR≤0.01 and $|\log_2 FC| \geq 1.5$) reported by each method and their intersections. Numbers underlined correspond to ORFs detected by at least three methods, which were declared as differentially expressed.

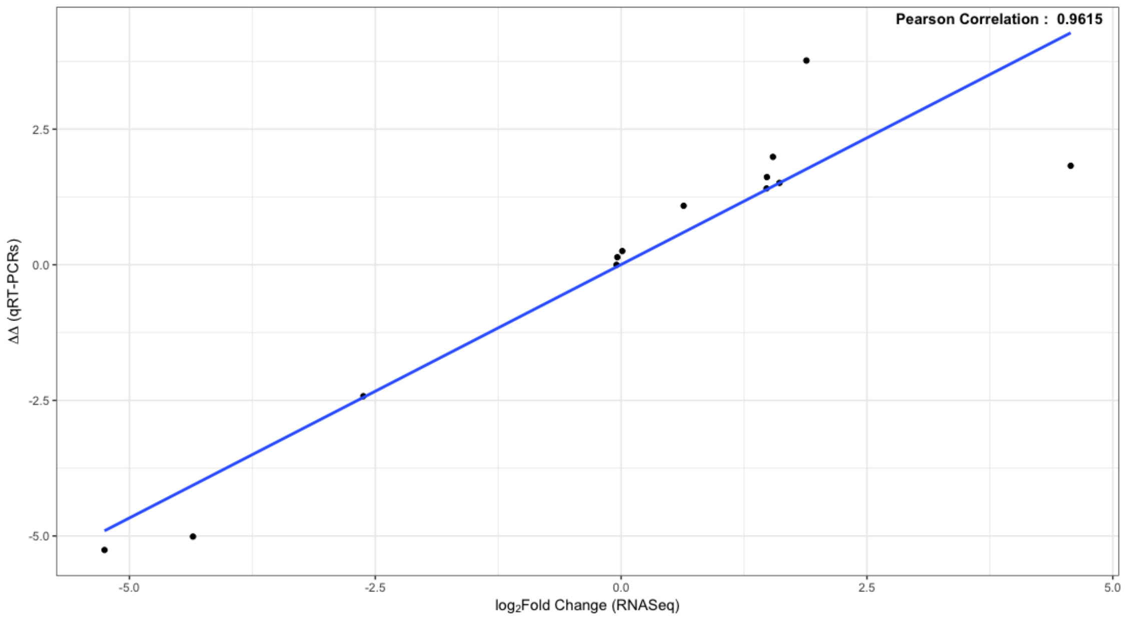

Figure S7. Correlation between qRT-PCR and RNA-Seq expression values of thirteen genes.

1.2 Supplementary Tables

Table S1. ORFs of *Klebsiella* sp. strain AqSCr encoding putative chromate reductases and their transcription under Cr(VI)-exposed conditions versus control conditions as determined by RNA-Seq

ORF/contig/family	Log$_2$FC	Protein sequence identity %	Reference enzymes	Accession # of reference enzymes	Microorganism (References)
BHE81_22565/ MJDM01000006.1/PF03358	-0.6	84.57	Soluble NAD(P)H-dependent FMN reductase (YieF)	ABJ74146	*Escherichia coli* K12 (Ackerley et al., 2004).
		60.96	Soluble NAD(P)H-dependent FMN reductase (YieF)	D5QFC5	*Gluconacetobacter hansenii* ATCC23769 (Jin et al., 2012).
		39.56	Soluble NAD(P)H-dependent FMN reductase (YieF)	AF375642	*Pseudomonas putida* (Park et al., 2000).
		34.08	Soluble NAD(P)H-dependent FMN reductase (YieF)	YP_917833	*Paracoccus denitrificans* (Mazoch et al., 2004).
BHE81_07500/ MJDM01000005.1/ PF00724	0.42	86.58	Soluble N-ethylmaleimide reductase (NemA)	P77258	*Escherichia coli* K12 substr. MG1655 (Robins et al., 2013)
BHE81_27485/ MJDM01000045.1 (plasmid)/	0.46	42.38			

PF00724					
BHE81_06255/ MJDM01000005.1/ PF00724	0	51.15	Soluble FMN reductase (ChrR)	B0JDW3	*Thermus scotoductus* (Opperman et al., 2008).
BHE81_01995/ MJDM01000003.1/ PF07992 and PF02852	-0.43	43.83	Membrane-bound Dihydrolipoamide dehydrogenase	P85207	*Thermus scotoductus* SA-01 (Opperman and van Heerden, 2008).
BHE81_01240/ MJDM01000001.1/ PF07992 and PF02852	0.63	43.23			
BHE81_20240/ MJDM01000005.1/ PF00881	-0.15	83.75	Soluble Oxygen-insensitive NADPH-dependent nitroreductase (NfsA).	P17117	*Escherichia coli* K12 (Ackerley et al., 2004).
		51.67	Soluble FMN-dependent reductase nitroreductase (NfsA)	P94424	*Bacillus subtilis* 168 (Morokutti et al., 2005).
		38.33	Soluble NADPH-dependent oxidoreductase (NfsA)	AAA21331	*Vibrio harveyi* KCTC 2720 (Kwak et al., 2003).
BHE81_27795/MJDM01000045.1 (plasmid)/ PF03358	0.15	67.96	Soluble NADPH-dependent reductase (ArsH)	ALJ68052	*Synechocystis sp.* PCC 6803 (Xue et al., 2014).

Table S2. Oligonucleotides used to perform knockout of genes encoding putative soluble chromate reductases and the ChrA transporter

Name	Sequence 5´→ 3´	Target ORF
396kpn1	tgcctgaaatcatcaacgaa	**BHE81_22565**
396kpn2	gacatgattgctcctttgg	
396kpn3	ccaaaggagcaatcatgtcagtgtaggctggagctgcttc	
396kpn4	atgctttaacccgccgaataattccggggatccgtcgac	
396kpn5	attcggcgggttaaagcat	
396kpn6	tccttcatcggtacctcgtc	
2515kpn1	ataactacgccgccattcc	**BHE81_06255**
2515kpn2	taagcgctccgctattcg	
2515kpn3	cgaatagcggagcgcttaagtgtaggctggagctgcttc	
2515kpn4	ggctacctgctggcgaataattccggggatccgtcgac	
2515kpn5	attcgccagcaggtagcc	
2515kpn6	agcagtaactcgcggtgttt	

2917kpn1	cttgatacctggcgcagtca	**BHE81_20240**
2917kpn2	cacgcctctccggttgata	
2917kpn3	tatcaaccggagaggcgtgaattccggggatccgtcgac	
2917kpn4	gatagcggttctcatgcaccagtgtaggctggagctgcttc	
2917kpn5	ggtgcatgagaaccgctatc	
2917kpn6	ggatcaccgcatcaaagtgt	
4253kpn1	cctgctggcctactatcagc	**BHE81_07500**
4253kpn2	gcttccgacatagtgactcctg	
4253kpn5	acaccgattacccgactctgt	
4253kpn6	ttgtaaccgtccggatcttc	
4253kpn3	caggagtcactatgtcggaagcaattccggggatccgtcgac	
4253kpn4	acagagtcgggtaatcggtgtagtgtaggctggagctgcttc	
266kpn1	ttccacgttaaaagggcatt	**BHE81_27485**
266kpn2	aaacatctgatgggtggcaat	
266kpn3	attgccacccatcagatgtttaattccggggatccgtcgac	
266kpn4	cagagaaccgcgaccgtagtgtaggctggagctgcttc	
266kpn5	acggtcgcggttctctg	
266kpn6	agcgaccttcggtgatactg	
ars1kpn1	tggtgtttatcactccttcacg	**BHE81_27795**
ars1kpn2	cagcacggacgtaagttcac	
ars1kpn3	gtgaacttacgtccgtgctgagtgtaggctggagctgcttc	
ars1kpn4	atggatttaaacactgctgcaaaattccggggatccgtcgac	
ars1kpn5	ttgcagcagtgtttaaatccat	
arsk1pn6	gcgtttcttaaaacacccgtta	
chrAkpn1	tgcgtaaaattcagcgtgac	**BHE81_07815**
chrAkpn2	gtcactctgcggaagaacg	
chrAkpn3	cgttcttccgcagagtgacagtgtaggctggagctgcttc	
chrAkpn4	cagcttccacttatagcgaaacaattccggggatccgtcgac	
chrAkpn5	gtttcgctataagtggaagctg	
chrAkpn6	tttttccgattaccgtgagg	

Table S3. Oligonucleotides used in qRT-PCRs

ORF	Sequences 5´→ 3´	Fragment size (bp)
sulP (BHE81_25425)	ctgggttccttctccctctt	103
	cgggaaccagcgagataa	
rpoE (BHE81_12570)	tggtagtgcgctatcagcat	101
	ccatgccttaacgaacgatt	
gltA2 (BHE81_19460)	ttcacatcaacggcttcttg	104

	gattcggtcgctaactggtc	
cybB (BHE81_07325)	atcggcatggtgatgatgta	98
	gtatccacccgctcaaaatc	
cybC (BHE81_25140)	gttagcgatgctggctgtct	102
	accacttgcaggttttctgc	
recN (BHE81_12780)	accatgaccgtctggatctc	98
	cggcatcacctgatgtttac	
lpdA (BHE81_01240)	gctaacaccctggaagtgga	103
	ggataaacggcagctgaatc	
pflB (BHE81_12590)	gctgctggatagcgaaaaag	98
	cgatttcacccagtttgctt	
cydB (BHE81_19515)	accaggtgtggcttatcacc	100
	accagaatcatcgccacatag	
gltA1 (BHE81_03385)	ggatcgatacggaaaacagc	99
	tcagcatgatgtaggccact	
rpoB (BHE81_23755)	gaaaccaactccgaaaccaa	104
	acggtcaggatcatccactc	
alaRS (BHE81_13165)	gagctgaacggcaaggttac	104
	tagaacggggtctggtcaag	
sdaAB (BHE81_09265)	gctggcctactgcaaagaga	102
	ttggcgaaataatcctcgat	

Table S4. One-way ANOVA test of reduction of 100µM of Cr(VI) after 24 hours by *Klebsiella* sp. strain AqSCr in aerobic LB medium at different pH (from Figure S3).				
	P-values resulting from paired comparisons			
	pH 7.5	pH 8.0	pH 8.5	pH 9.0

pH 7.0	1.86E-01	**3.56E-02**	**2.35E-03**	**1.39E-03**
pH 7.5		2.17E-01	**4.15E-03**	**2.13E-03**
pH 8.0			**3.05E-03**	**1.91E-03**
pH 8.5				5.24E-02

Analysis conducted with data presented in the Figure S3.
A p-value < 0.005 was considered statistically significant (Bold).

Table S5. Selected genes of *Klebsiella* sp. strain AqSCr upregulated in the presence of 11 mM Cr(VI)

ORF	Gene product	Gene name	KO	Log_2FC	p-adj
**Oxidative stress**					
BHE81_13040	Alkylhydroperoxidase	*ahp*		3.10	2.99E-03
BHE81_06815	Alkylhydroperoxidase AhpD family core domain protein	*ahpD*		3.97	7.12E-11
BHE81_04165	Lipid hydroperoxide peroxidase/ Thiol peroxidase Tpx-type	*tpx*	K11065	0.98	4.03E-02
BHE81_24245	Transcriptional regulator/ Regulatory protein	*soxS*	K13631	1.74	1.23E-02
BHE81_07325	Cytochrome b_{561}	*cybB*	K12262	1.88	7.46E-03
BHE81_23030	Manganese superoxide dismutase	*sodA*	K04564	1.74	1.58E-04
BHE81_06560	Pyrroloquinoline-quinone synthase	*pqqC*	K06137	2.73	2.91E-03
**Iron-sulfur cluster assembly**					
BHE81_12370	Chaperone HscB	*hscB*	K04082	1.76	7.05E-04
BHE81_12375	Iron-sulfur cluster assembly Iron binding protein	*iscA*	K13628	1.79	1.05E-05
BHE81_12385	Cysteine desulfurase IscS subfamily	*iscS*	K04487	2.29	5.90E-10
BHE81_12390	Iron-sulfur cluster assembly transcriptional regulator	*iscR*	K13643	1.72	5.42E-08
BHE81_08295	Cysteine desulfurase SufS subfamily	*sufS*	K11717	2.07	1.31E-07
BHE81_08300	Iron-sulfur cluster assembly protein	*sufD*	K09015	2.15	4.04E-07
BHE81_08310	Iron-sulfur cluster assembly protein	*sufB*	K09014	2.71	1.14E-09
BHE81_08315	Iron-sulfur cluster assembly iron binding protein	*sufA*	K05997	2.49	1.99E-03
**DNA repair and replication**					
BHE81_26455	Single-stranded DNA-binding protein	*ssb*	K03111	1.90	9.75E-03
BHE81_10860	Deoxyribonuclease IV	*nfo*	K01151	1.75	3.42E-03
BHE81_12780	DNA repair protein	*recN*	K03631	1.54	2.95E-07
BHE81_03730	Deoxyribonuclease, exonuclease	*cho*	K05984	1.51	1.07E-02
BHE81_21170	DinI like family	*dinI*		1.77	4.40E-05
BHE81_21980	DinI like family	*dinI*		4.90	7.56E-05
BHE81_08655	DNA-binding protein H-NS	*hns*	K03746	1.81	1.42E-04
BHE81_13175	RecA protein	*recA*	K03553	1.23	1.22E-04
BHE81_13045	Glutaredoxin-like protein	*nrdH*	K06191	5.16	1.66E-03

BHE81_13055	Ribonucleotide reductase of class Ib alpha subunit	*nrdA*	K00525	3.40	2.39E-11
BHE81_13060	Ribonucleotide reductase of class Ib beta subunit	*nrdB*	K00526	2.53	2.01E-04
**Sulfate metabolism**					
BHE81_25425	Sulfate permease	*sulP*		4.57	7.28E-13
BHE81_25430	Carbonic anhydrase	*can*	K01673	6.43	3.52E-11
BHE81_20670	Alkanesulfonates ABC transporter ATP-binding protein	*ssuB*	K15555	4.07	1.90E-21
BHE81_20675	Alkanesulfonate transporter permease subunit	*ssuC*	K15554	4.07	1.26E-14
BHE81_20680	Alkanesulfonate monooxygenase	*ssuD*	K04091	3.90	6.84E-10
BHE81_20685	Alkanesulfonate transporter substrate-binding protein	*ssuA*	K15553	5.23	4.80E-13
BHE81_20690	NAD(P)H-dependent FMN reductase	*ssuE*	K00299	3.89	1.14E-09
BHE81_13785	Alkanesulfonate monooxygenase	*ssuD*	K04091	2.66	2.64E-03
BHE81_13795	Alkanesulfonate transporter substrate-binding protein	*ssuA*	K15553	4.18	1.51E-15
BHE81_08155	Alkanesulfonate monooxygenase	*ssuD*	K04091	3.00	5.39E-05
BHE81_04410	Methionine ABC transporter substrate-binding protein	*metQ*	K02073	6.06	5.82E-05
BHE81_04415	Methionine ABC transporter ATP-binding protein	*metN*	K02071	3.18	5.66E-04
BHE81_04420	Methionine ABC transporter permease subunit	*metI*	K02072	2.70	2.29E-03
BHE81_05670	Methionine ABC transporter ATP-binding protein	*metN*	K02071	5.57	3.57E-06
BHE81_05675	Methionine ABC transporter substrate-binding protein	*metQ*	K02073	4.59	1.50E-06
BHE81_02450	Taurine-binding periplasmic protein	*tauA*	K15551	2.92	1.17E-04
BHE81_02455	Taurine transporter system ATP-binding protein	*tauB*	K10831	2.50	3.28E-03
BHE81_02460	Taurine transport system permease protein	*tauC*	K15552	1.61	5.09E-01
BHE81_02465	Taurine dioxygenase	*tauD*	K03119	2.57	1.70E-04
BHE81_02200	Arylsulfatase		K01130	2.78	1.22E-03
**Phosphate uptake**					
BHE81_21040	Phosphate starvation-inducible protein	*phoH*	K06217	2.32	1.43E-09
BHE81_02595	Phosphate regulon transcriptional regulatory protein	*phoB*	K07657	2.33	6.86E-04
BHE81_02600	Two-component system sensor histidine kinase	*phoR*	K07636	1.33	3.36E-03
BHE81_22625	Phosphate ABC transporter periplasmic phosphate-binding protein	*ptsS*	K02040	2.90	3.00E-12
**Molybdenum uptake**					
BHE81_19645	Molybdenum ABC transporter periplasmic molybdenum-binding protein	*modA*	K02020	1.63	4.15E-04
BHE81_19650	Molybdenum ABC transporter permease protein	*modB*	K02018	1.92	2.88E-07
BHE81_19655	molybdenum ABC transporter ATP-binding protein	*modC*	K02017		
**Iron uptake and homeostasis**					
BHE81_15085	TonB system transport protein ExbD	*exbD*	K03559	2.43	3.51E-05
BHE81_15090	TonB-system energizer ExbB	*exbB*	K03561	2.21	1.03E-07

BHE81_26615	RNA polymerase sigma factor	*fecI*	K03088	3.06	4.97E-06
BHE81_26625	Fe(III)-dicitrate transporter protein	*fecA*	K16091	2.59	1.16E-05
BHE81_26630	Fe(III)-dicitrate transporter iron-binding subunit	*fecB*	K02016	2.67	2.00E-07
BHE81_26635	Fe(III)-dicitrate transporter permease subunit	*fecC*	K02015	4.29	1.30E-06
BHE81_26640	Fe(III)-dicitrate transporter permease protein	*fecD*	K02015	1.75	3.56E-03
BHE81_26645	Fe(III)-dicitrate transporter ATP-binding protein	*fecE*	K02013	3.97	9.69E-04
BHE81_08220	Fe(III) ABC transporter substrate-binding protein		K02016	3.47	9.11E-06
BHE81_08225	Fe(III)-siderophore ABC transporter permease subunit	*feC1*	K02015	2.47	2.30E-04
BHE81_08230	Fe(III)-siderophore ABC transporter ATP-binding protein	*feE1*	K02013	2.01	3.44E-03
BHE81_08360	Fe(III) ABC transporter periplasmic substrate-binding protein		K02016	2.50	3.95E-05
BHE81_08365	Fe(III) ABC transporter permease subunit	*feC2*	K02015	3.05	1.21E-04
BHE81_08370	Fe(III) ABC transporter ATP-binding protein	*feE2*	K02013	2.36	1.67E-03
BHE81_13465	TonB-dependent hemin ferrichrome receptor	*hemR*	K16087	4.00	2.96E-12
BHE81_08445	Hemin uptake protein	*hemP*		1.93	8.56E-03
BHE81_13470	Hemin transport protein	*hmuS*	K07225	3.58	1.07E-08
BHE81_13475	Hemin ABC transporter substrate-binding protein	*hmuT*	K02016	4.65	6.91E-07
BHE81_13480	Hemin ABC transporter permease subunit	*hmuU*	K02015	4.09	5.43E-09
BHE81_13485	Hemin ABC transporter ATP-binding protein	*hmuV*	K02013	4.99	5.82E-05
BHE81_18780	TonB-dependent ferric enterobactin receptor	*fepA*	K19611	4.12	3.61E-11
BHE81_18785	Enterobactin esterase	*fes*	K07214	3.41	5.03E-08
BHE81_18795	Enterobactin synthetase	*entF*	K02364	2.60	2.66E-06
BHE81_18800	Fe(III)-enterobactin ABC transporter ATP-binding protein	*fepC*	K02013	3.53	7.15E-06
BHE81_18805	Fe(III)-enterobactin ABC transporter permease subunit	*fepG*	K02015	2.00	9.13E-03
BHE81_18815	Enterobactin MFS exporter	*entS*	K08225	4.50	6.47E-09
BHE81_18820	Fe(III)-enterobactin ABC transporter substrate-binding protein	*fepB*	K02016	3.19	1.86E-03
BHE81_18825	Isochorismate synthase of siderophore biosynthesis	*entC*	K02361	4.12	7.82E-11
BHE81_18830	2,3-dihydroxybenzoate-AMP ligase of siderophore biosynthesis	*entE*	K02363	4.74	8.69E-21
BHE81_18835	Isochorismatase of siderophore biosynthesis	*entB*	K01252	4.57	5.90E-11
BHE81_18840	2,3-dihydro-2,3-dihydroxybenzoate dehydrogenase siderophore	*entA*	K00216	3.33	2.79E-06
BHE81_18845	Proofreading thioesterase in enterobactin biosynthesis	*entH*		2.44	4.16E-05
BHE81_01495	Ferric hydroxamate outer membrane receptor	*fhuA1*	K02014	2.59	6.62E-05
BHE81_26060	TonB-dependent Fe(III) receptor	*fhuA2*	K02014	1.58	2.08E-03
BHE81_03560	Ferrichrome transporter ATP-binding protein	*fhuC*	K02013	1.84	8.56E-03
BHE81_03565	Fe(III)-siderophore ABC transporter permease	*fhuB*	K02015	2.08	3.08E-03
BHE81_03570	Fe(III) ABC transporter substrate-binding protein	*fhuD*	K02016	1.96	4.53E-03
BHE81_10835	Catecholate siderophore receptor	*cirA*	K16089	2.57	1.78E-07

BHE81_15690	NADPH-dependent ferric siderophore reductase	*yqjH*	K07229	2.04	7.89E-05
BHE81_16810	Bacterioferritin-associated ferredoxin	*bfd*	K02192	5.30	2.26E-03
BHE81_09125	Ferrioxamine B receptor	*foxA*	K02014	2.25	7.58E-07
BHE81_13420	Manganese/iron ABC transporter periplasmic-binding protein	*sitA*	K11604	3.99	2.73E-23
BHE81_13425	Manganese/iron ABC transporter ATP-binding protein	*sitB*	K11607	4.53	7.41E-17
BHE81_13430	Manganese/iron ABC transporter inner membrane permease protein	*sitC*	K11605	4.63	2.28E-10
BHE81_13435	Manganese/iron ABC transporter inner membrane permease protein	*sitD*	K11606	4.35	4.20E-11
**Manganese uptake**					
BHE81_11640	Hypothetical protein	*mntX*		1.88	5.68E-04
BHE81_11645	Manganese transport protein	*mntH*	K03322	2.24	9.54E-08
BHE81_13420	Manganese ABC transporter periplasmic-binding protein	*sitA*	K11604	3.99	2.73E-23
BHE81_13425	Manganese ABC transporter 2C ATP-binding protein	*sitB*	K11607	4.53	7.41E-17
BHE81_13430	Manganese ABC transporter inner membrane permease protein	*sitC*	K11605	4.63	2.28E-10
BHE81_13435	Manganese ABC transporter inner membrane permease protein	*sitD*	K11606	4.35	4.20E-11
**Envelope stress response**					
BHE81_12570	RNA polymerase sigma factor	*rpoE*	K03088	1.61	2.41E-05
BHE81_03725	ATP-independent periplasmic protein-refolding chaperone	*spy*		4.70	7.14E-34
BHE81_23050	Stress adaptor protein	*cpxP*	K06006	2.02	8.88E-05
BHE81_09355	Heat shock protein	*htpX*	K03799	2.25	2.75E-10
BHE81_01550	Serine endoprotease/chaperone protein	*htrA*	K04771	3.34	8.66E-39
BHE81_14840	Membranal heat shock associated protein	*yggG*	K07387	1.95	1.56E-05
**Osmotic stress**					
BHE81_10710	Osmoprotectant ABC transporter substrate-binding protein	*osmF*	K05845	1.51	5.38E-04
BHE81_13065	L-proline glycine betaine ABC transport system permease protein	*proV*	K02000	2.14	1.09E-02
BHE81_04010	Osmotically inducible lipoprotein B precursor	*osmB*	K04062	3.99	2.97E-07
BHE81_00470	Osmotically inducible protein	*osmY*	K04065	2.47	5.87E-10
BHE81_27505	Osmotically inducible protein	*osmC*	K04063	2.44	5.42E-04
BHE81_13545	RNA polymerase sigma factor	*rpoS*	K03087	0.99	1.96E-02
BHE81_08665	Two-component system response regulator	*rssB*	K02485	1.69	4.51E-03
BHE81_07005	ABC transporter substrate-binding protein		K02030	5.70	7.55E-08
BHE81_07010	Ectoine/hydroxyectoine ABC transporter ATP-binding protein	*ehuA*	K02028	7.42	1.25E-06
BHE81_07020	Amino acid ABC transporter permease		K02029	5.68	1.44E-05
**Fatty acid metabolism**					
BHE81_11550	Long-chain fatty acid transporter	*fadL*	K06076	1.60	9.92E-05

BHE81_13000	Fe(2+)/alpha-ketoglutarate-dependent dioxygenase	*lpxO*	K12979	1.84	8.82E-04
BHE81_09095	Fatty acid desaturase	*des*		5.41	2.27E-04
BHE81_24760	Phosphatidylserine decarboxylase	*psd*	K01613	1.60	4.38E-05
BHE81_08150	Butyryl-CoA dehydrogenase	*bcd*	K00248	4.04	2.51E-05
BHE81_14340	Acetyl-CoA acetyltransferase	*atoB*	K00626	2.02	1.74E-03
BHE81_01820	Butyryl-CoA dehydrogenase	*fadE*	K06445	2.61	1.84E-04
BHE81_23670	3-ketoacyl-CoA thiolase	*fadA*	K00632	2.37	1.43E-07
BHE81_20740	3-hydroxyacyl-[acyl-carrier-protein] dehydratase	*fabA*	K01716	0.89	
**Ribosome related**					
BHE81_15345	30S ribosomal protein S21	*rpsU*	K02970	2.34	2.01E-04
BHE81_16205	50S ribosomal protein L27	*rpmA*	K02899	2.17	2.40E-04
BHE81_16380	30S ribosomal protein S9	*rpsI*	K02996	1.67	4.95E-03
BHE81_16385	50S ribosomal protein L13	*rplM*	K02871	1.77	3.86E-03
BHE81_18145	50S ribosomal protein L28	*rpmB*	K02902	1.95	1.82E-07
BHE81_06740	30S ribosomal protein S22	*sra*		1.71	6.27E-03
**Energy metabolism**					
BHE81_24330	Acetyl-coenzyme A synthetase	*acs*	K01895	2.34	8.18E-12
BHE81_19460	Citrate synthase (si)	*gltA2*	K01647	1.48	5.44E-05
BHE81_19465	Succinate dehydrogenase cytochrome b_{556} subunit	*sdhC*	K00241	1.76	4.13E-02
BHE81_19470	Succinate dehydrogenase hydrophobic membrane anchor protein	*sdhD*	K00242	1.68	2.02E-03
BHE81_19475	Succinate dehydrogenase flavoprotein subunit	*sdhA*	K00239	1.52	5.24E-03
BHE81_19480	Succinate dehydrogenase iron-sulfur protein	*sdhB*	K00240	0.84	3.29E-01
BHE81_07325	Cytochrome b_{561}	*cybB*	K12262	1.88	7.46E-03
BHE81_25140	Soluble cytochrome b_{562}	*cybC*	K15536	1.48	2.87E-04
BHE81_22675	ATP synthase delta chain	*atpH*	K02113	1.81	1.78E-07
BHE81_22680	ATP synthase F0 sector subunit b	*atpF*	K02109	1.62	2.75E-05
BHE81_22685	ATP synthase F0 sector subunit c	*atpE*	K02110	1.71	6.45E-04
BHE81_22695	ATP synthase protein I	*atpI*	K02116	1.69	1.36E-03

Genes considered differentially expressed with a $|\log_2(\text{fold change, FC})| \geq 1.5$ and adjusted *p*-value (p-adj) ≤ 0.01 detected at least three methods, $\log_2$FC and p-adj values were calculated by edgeR (See Materials and Methods for details). Genes underlined are those that were found upregulated but outside of the cutoff parameters. Gene annotation was manually curated. KEGG orthology (KO) identifiers were assigned using GhostKOALA from KEGG.

Table S6. Selected genes of *Klebsiella* sp. strain AqSCr downregulated in presence of 11 mM Cr(VI)

ORF	Gene product	Gene name	KO	Log$_2$FC	p-adj
**Ribosomal functions**					
BHE81_25675	Hydroxylase YcfD of 50S ribosomal protein L16	*ycfD*	K18850	-4.05	2.20E-14
BHE81_03455	Lysyl-tRNA synthetase	*lysS*	K04567	-2.25	2.19E-04

**Sulfur metabolism**					
BHE81_03655	Thiosulfate sulfurtransferase	*tst*	K01011	-3.57	2.95E-13
**Envelope stress response**					
BHE81_19320	N-acetylglucosamine-regulated outer membrane porin	*omp-nag*		-2.34	4.03E-05
BHE81_10030	Outer membrane protein N precursor	*ompN*	K14062	-2.03	8.13E-04
BHE81_03840	Outer membrane protein W precursor	*ompW*	K07275	-5.18	4.33E-29
BHE81_20620	Outer membrane protein F precursor	*ompF*	K09476	-3.73	3.98E-21
BHE81_18315	Aquaporin/Glycerol uptake facilitator protein GLPF	*glpF*	K02440	-1.85	1.74E-05
**General and oxidative stress**					
BHE81_06605	Catalase/peroxidase HPI	*katG*	K03782	-2.84	1.54E-10
BHE81_07530	Fe-Superoxide dismutase	*sodB*	K04564	-3.17	7.64E-13
BHE81_19080	Universal stress protein G	*upsG*	K11932	-1.81	2.01E-04
BHE81_04460	Universal stress protein F	*upsF*	K14061	-3.62	2.14E-11
BHE81_04980	Universal stress protein E	*upsE*	K14055	-1.69	5.23E-04
**Energy**					
BHE81_16965	Nitrite reductase large subunit	*nir*	K00362	-6.89	1.13E-21
BHE81_06840	Formate dehydrogenase N subunit gamma	*fdnI*	K08350	-9.15	2.56E-10
BHE81_06845	Formate dehydrogenase N subunit beta	*fdnH*	K08349	-7.41	2.24E-17
BHE81_06850	Formate dehydrogenase N subunit alpha	*fdnG*		-7.18	1.10E-20
BHE81_08705	Respiratory nitrate reductase gamma chain	*narI*	K00374	-8.79	7.80E-07
BHE81_08710	Respiratory nitrate reductase delta chain	*narJ*	K00373	-8.13	2.33E-30
BHE81_08715	Respiratory nitrate reductase beta chain	*narH*	K00371	-7.87	2.39E-42
BHE81_08720	Respiratory nitrate reductase alpha chain	*narG*	K00370	-8.36	5.82E-85
BHE81_08730	Nitrate/nitrite transporter	*ntr*	K02575	-7.82	1.72E-45
BHE81_08735	Two-component system sensor histidine kinase	*narX*	K07673	-2.30	6.35E-07
BHE81_08740	Two-component system response regulator	*narL*	K07684	-2.49	6.57E-10
BHE81_17935	Electron transport protein	*hydN*	K05796	-3.42	7.56E-07
BHE81_19510	Cytochrome d terminal oxidase subunit I	*cydA*	K00425	-2.63	2.35E-11
BHE81_19515	Cytochrome d ubiquinol oxidase subunit II	*cydB*	K00426	-2.62	8.45E-16
BHE81_19520	Cyd operon protein	*ybgT*		-2.37	1.41E-03
BHE81_19525	Cyd operon protein	*ybgE*		-1.78	9.14E-04
BHE81_20465	Dimethyl sulfoxide reductase subunit A	*dmsA1*	K07306	-4.79	1.10E-20
BHE81_20470	Dimethyl sulfoxide reductase subunit B	*dmsB1*	K07307	-3.61	4.12E-08
BHE81_20475	Dimethyl sulfoxide reductase subunit C	*dmsC1*	K07308	-3.07	2.35E-06
BHE81_05240	Dimethyl sulfoxide reductase subunit B	*dmsB2*	K07307	-2.11	5.98E-05
BHE81_05245	Dimethyl sulfoxide reductase subunit B	*dmsB3*	K07307	-3.69	1.18E-04
BHE81_05250	Dimethyl sulfoxide reductase subunit A	*ynfE*	K07309	-2.54	2.76E-05
BHE81_24725	Fumarate reductase subunit D	*frdD*	K00247	-3.80	1.74E-10
BHE81_24730	Fumarate reductase subunit C	*frdC*	K00246	-4.20	5.82E-15
BHE81_24735	Fumarate reductase iron-sulfur subunit	*frdB*	K00245	-3.93	6.43E-14
BHE81_24740	Fumarate reductase flavoprotein subunit	*frdA*	K00244	-4.41	6.25E-28
BHE81_03385	Citrate (Si)-synthase	*gltA1*	K01647	-4.35	3.79E-11
BHE81_13360	[NiFe] hydrogenase metallocenter assembly protein	*hypD*	K04654	-4.22	1.25E-10

BHE81_13365	[NiFe] hydrogenase metallocenter assembly protein	*hypE*	K04655	-4.20	2.36E-19
**Menaquinone biosynthesis**					
BHE81_11200	O-succinylbenzoate-CoA ligase	*menE*	K01911	-1.94	1.10E-05
BHE81_11205	O-succinylbenzoate synthase	*menC*	K02549	-2.40	4.50E-09
BHE81_11210	Naphthoate synthase	*menB*	K01661	-3.14	1.69E-11
BHE81_11215	2-succinyl-6-hydroxy-2, 4-cyclohexadiene-1-carboxylate synthase	*menH*	K08680	-2.13	2.32E-04
BHE81_11220	2-succinyl-5-enolpyruvyl-6-hydroxy-3- cyclohexene-1-carboxylic-acid synthase	*menD*	K02551	-2.76	5.20E-08
BHE81_11225	Menaquinone-specific isochorismate synthase	*menD*	K02552	-2.24	2.01E-06
BHE81_20940	NAD(P)H:quinone oxidoreductase	*wrbA*	K03809	-1.61	1.20E-04
**Lipid metabolism**					
BHE81_03650	Putative phosphatidylglycerophosphate synthase	*pgsA*		-2.60	1.29E-04
BHE81_11130	Glycerophosphoryl diester phosphodiesterase periplasmic	*glpQ*	K01126	-2.62	5.72E-07
BHE81_11135	Glycerol-3-phosphate transporter	*glpT*	K02445	-4.14	8.70E-20
BHE81_11140	Glycerol-3-phosphate dehydrogenase subunit A	*glpA*	K00111	-7.03	7.64E-07
BHE81_11145	Glycerol-3-phosphate dehydrogenase subunit B	*glpB*	K00112	-6.87	3.43E-29
BHE81_11150	Glycerol-3-phosphate dehydrogenase subunit C	*glpC*	K00113	-4.86	3.71E-11
BHE81_15670	Phosphoenolpyruvate-glycerone phosphotransferase subunit	*dhaK*	K05878	-3.22	5.39E-05
BHE81_15675	Phosphoenolpyruvate-dihydroxyacetone phosphotransferase ADP-binding subunit	*dhaL*	K05879	-3.86	4.92E-09
BHE81_15680	Phosphoenolpyruvate-dihydroxyacetone phosphotransferase subunit	*dhaM*	K05881	-2.36	8.65E-03
BHE81_17170	Glycerol-3-phosphate dehydrogenase	*glpA*	K00111	-3.24	2.62E-23
BHE81_15660	Glycerol dehydrogenase	*gldA*	K00005	-1.72	7.91E-03
BHE81_18320	Glycerol kinase	*glpK*	K00864	-2.11	1.89E-09
**Cobalt transport and cobalamin synthesis**					
BHE81_13895	Adenosylcobinamide-phosphate guanylyltransferase	*cobP*	K02231	-2.22	7.44E-03
BHE81_13900	Cobyric acid synthase	*cobQ*	K02232	-2.08	3.21E-03
BHE81_13905	ATPase component of energizing module of cobalt ECF transporter	*cbiO*	K02006	-1.76	2.43E-02
BHE81_13910	Transmembrane component of energizing module of cobalt ECF transporter	*cbiQ*	K02008	-3.47	9.94E-05
BHE81_13920	Substrate-specific component of cobalt ECF transporter	*cbiM*	K02007	-3.91	2.54E-06
BHE81_13925	Cobalt-precorrin-2 C20-methyltransferase	*cbiL*	K03394	-3.42	9.92E-05
BHE81_13930	Sirohydrochlorin cobaltochelatase	*cbiK*	K02190	-3.35	1.04E-01
BHE81_13935	Cobalt-precorrin-6x reductase	*cbiJ*	K05895	-4.55	4.94E-07
BHE81_13940	Cobalt-precorrin-3b C17-methyltransferase	*cbiH*	K05934	-4.65	6.50E-12
BHE81_13945	Cobalamin biosynthesis protein	*cbiG*	K02189	-4.88	1.56E-13
BHE81_13950	Cobalt-precorrin-4 C11-methyltransferase	*cbiF*	K05936	-5.73	5.29E-09
BHE81_13955	Cobalt-precorrin-6y C15-methyltransferase	*cbiT*	K02191	-6.30	3.01E-10
BHE81_13960	Cobalt-precorrin-6y C5-methyltransferase	*cbiE*	K03399	-4.67	2.92E-09
BHE81_13965	Cobalt-precorrin-6 synthase2C anaerobic	*cbiD*	K02188	-5.45	4.90E-24
BHE81_13970	Cobalt-precorrin-8x methylmutase	*cbiC*	K06042	-5.53	6.31E-22
BHE81_13975	Adenosylcobinamide-phosphate synthase	*cbiB*	K02227	-4.96	4.41E-29
BHE81_13980	Cobyrinic acid a,c-diamide synthase	*cbiA*	K02224	-4.55	5.30E-26

BHE81_13985	Propanediol utilization transcriptional activator			-3.42	2.02E-03
**Iron II acquisition**					
BHE81_03845	Iron-uptake factor	*piuC*	K07336	-3.49	5.42E-08
BHE81_17105	Ferrous iron transport protein B	*feoB*	K04759	-2.03	1.41E-07
**Nickel acquisition**					
BHE81_17475	Nickel ABC transporter periplasmic nickel-binding protein	*nikA*	K15584	-2.80	4.17E-05
BHE81_17495	Nickel transport ATP-binding protein	*nikD1*		-2.61	4.60E-06
BHE81_17500	Nickel transport ATP-binding protein	*nikD2*		-2.75	3.46E-04
**Phage related genes**					
BHE81_15380	Phage tail tape measure protein	*phageA*		-2.04	3.78E-03
BHE81_15385	Phage tail protein	*phageB*		-3.80	1.68E-03
BHE81_15395	Phage major tail tube protein	*phageC*	K06908	-3.70	3.52E-11
BHE81_15400	Phage tail protein	*phageD*	K06907	-4.56	9.82E-14
BHE81_15410	Phage tail fiber	*phageE*		-2.28	1.86E-04
BHE81_15425	Phage baseplate assembly protein	*phageF*	K06903	-2.10	6.73E-03
BHE81_15430	Phage baseplate assembly protein V	*phageG*		-2.83	1.46E-03
BHE81_15435	Phage tail completion protein	*phageH*		-3.16	2.20E-03
BHE81_15440	Phage tail protein	*phageI*		-2.80	1.83E-03
BHE81_15475	Phage terminase endonuclease subunit	*phageJ*		-5.20	8.13E-09
BHE81_15480	Phage major capsid protein, P2 family	*phageK*		-4.63	5.74E-12
BHE81_15485	Phage capsid scaffolding protein	*phageL*		-3.22	4.10E-08
BHE81_21690	Phage portal protein	*phageM*		-3.03	1.75E-03
**DNA repair and replication**					
BHE81_21560	Exodeoxyribonuclease VIII	*recE*		-2.57	3.12E-05
BHE81_00985	Chromosome initiation inhibitor	*iciA*		-2.33	1.08E-02
BHE81_25210	Ribonucleotide reductase of class III activating protein	*nrdG*	K04068	-4.16	5.54E-10
BHE81_25215	Ribonucleotide reductase of class III large subunit	*rtprB*	K00527	-4.95	3.59E-37

Genes considered differentially expressed with a $|\log_2(\text{fold change, FC})| \geq 1.5$ and adjusted *p*-value (p-adj) ≤ 0.01 detected at least three methods, $\log_2$FC and p-adj values were calculated by edgeR (See Materials and Methods for details). Genes underlined are those that were found upregulated but outside of the cutoff parameters. Gene annotation was manually curated. KEGG orthology (KO) identifiers were assigned using GhostKOALA from KEGG.

Table S7. Genes differentially expressed harbored in plasmids

ORF	Gene product	Log_2FC	p-adj	Contig
BHE81_26105	Conjugal transfer protein TraT	-1.72	9.20E-03	MJDM01000014.1

BHE81_26455	Single-stranded DNA-binding protein	1.90	9.70E-03	MJDM01000040.1
BHE81_26615	RNA polymerase subunit sigma	3.06	5.00E-06	MJDM01000041.1
BHE81_26625	Transporter	2.59	1.00E-05	
BHE81_26630	Iron-dicitrate transporter substrate-binding subunit FecB	2.67	2.00E-07	
BHE81_26635	Iron-dicitrate transporter permease subunit FecC	4.29	1.00E-06	
BHE81_26640	Iron-dicitrate transporter subunit FecD	1.75	3.60E-03	
BHE81_26645	Ferric citrate ABC transporter ATP-binding protein FecE	3.97	1.00E-03	
BHE81_26675	Beta-D-galactosidase LacZ	-1.75	9.00E-05	
BHE81_26715	Hypothetical protein	2.56	3.00E-04	MJDM01000043.1
BHE81_26875	Hypothetical protein	-1.56	8.00E-05	MJDM01000045.1
BHE81_26900	Hypothetical protein	2.10	2.30E-03	
BHE81_27010	TetR family transcriptional regulator	1.87	6.80E-03	
BHE81_27280	Hypothetical protein	-2.28	8.80E-03	
BHE81_27505	Osmotically inducible protein C	2.44	5.00E-04	
BHE81_27600	Transposase	-2.44	2.00E-06	
BHE81_27725	IS66 family transposase	-2.58	7.00E-10	
BHE81_27730	Chlorite dismutase	-2.47	5.00E-13	
BHE81_27830	Type-F conjugative transfer system pilin acetylase TraX	-1.86	2.00E-04	

BHE81_26060	TonB-dependent copper receptor	1.58	2.10E-03	MJDM01000052.1
Genes considered differentially expressed with a $\lvert log_2$(fold change, FC)$\rvert \geq 1.5$ and adjusted *p*-value (p-adj) ≤ 0.01 detected at least three methods, log_2FC and p-adj values were calculated by edgeR (See Materials and Methods for details).				

Table S8. Top upregulated genes of strain AqSCr in the adapted state to 11 mM Cr(VI)

ORF	Gene product	Log_2FC	p-adj
BHE81_07035	Hydrolase	8.22	1.23E-09
BHE81_07010	Ectoine/hydroxyectoine ABC transporter ATP-binding protein EhuA	7.43	7.55E-08
BHE81_25430	Carbonic anhydrase	6.43	3.52E-11
BHE81_04410	Methionine-binding protein	6.06	5.82E-05
BHE81_02255	Hypothetical protein	6.06	6.10E-05
BHE81_07030	Monooxygenase	6.05	1.35E-07
BHE81_07025	ABC transporter substrate-binding protein	5.84	9.73E-07
BHE81_07005	ABC transporter substrate-binding protein	5.71	1.28E-06
BHE81_07015	Amino acid ABC transporter permease	5.69	1.25E-06
BHE81_05670	Methionine ABC transporter ATP-binding protein	5.57	3.57E-06
BHE81_09095	Fatty acid desaturase	5.41	2.27E-04
BHE81_16810	Bacterioferritin-associated ferredoxin	5.30	2.26E-03
BHE81_07040	Alkane 1-monooxygenase	5.24	7.48E-09

BHE81_07020	GNAT family N-acetyltransferase	5.24	1.44E-05
BHE81_20685	Aliphatic sulfonate ABC transporter substrate-binding protein	5.23	4.80E-13
BHE81_04625	2-(1,2-epoxy-1,2-dihydrophenyl)acetyl-CoA isomerase	5.17	1.76E-03
BHE81_13045	NrdH-redoxin	5.16	1.66E-03
BHE81_06805	Glutathione ABC transporter ATP-binding protein	4.99	1.39E-10
BHE81_13485	Heme ABC transporter ATP-binding protein	4.99	5.82E-05
BHE81_06795	Peptide ABC transporter permease	4.96	1.56E-06
BHE81_06810	Luciferase	4.94	1.14E-07
BHE81_21980	DNA-damage-inducible protein I	4.90	7.56E-05
BHE81_02725	Hypothetical protein	4.77	2.25E-03
BHE81_18830	2,3-dihydroxybenzoate-AMP ligase	4.74	8.69E-21
BHE81_03725	ATP-independent periplasmic protein-refolding chaperone	4.70	7.14E-34
BHE81_02385	Hypothetical protein	4.67	6.72E-26
BHE81_13475	Hemin ABC transporter substrate-binding protein	4.65	6.91E-07
BHE81_13430	Hypothetical protein	4.63	2.28E-10
BHE81_05675	Metal ABC transporter substrate-binding protein	4.59	1.50E-06
BHE81_18835	Isochorismatase	4.57	5.90E-11
BHE81_25425	Sulfate permease	4.57	7.28E-13

BHE81_19025	ABC transporter substrate-binding protein	4.53	4.15E-04
BHE81_13425	Manganese/iron transporter ATP-binding protein	4.53	7.41E-17
BHE81_18815	MFS transporter	4.50	6.47E-09
BHE81_20950	Stress-induced protein	4.48	4.77E-10
BHE81_13435	Iron ABC transporter permease	4.35	4.20E-11
BHE81_03490	Rhizopine-binding protein	4.31	2.42E-02
BHE81_24325	Methionyl-tRNA formyltransferase	4.29	9.87E-03
BHE81_26635	Ion-dicitrate transporter permease subunit	4.29	1.30E-06
BHE81_07635	Hypothetical protein	4.28	1.33E-09
BHE81_07215	Hypothetical protein	4.25	1.06E-04
BHE81_13795	AAA family ATPase	4.18	1.51E-15
BHE81_18780	Outer membrane receptor protein	4.12	3.61E-11
BHE81_18825	Isochorismate synthase EntC	4.12	7.82E-11
BHE81_06800	ABC transporter permease	4.11	3.86E-07
BHE81_13480	Iron ABC transporter	4.09	5.43E-09
BHE81_20670	Aliphatic sulfonates ABC transporter ATP-binding protein	4.07	1.90E-21
BHE81_20675	Alkanesulfonate transporter permease subunit	4.07	1.26E-14
BHE81_08150	Acyl-CoA dehydrogenase	4.04	2.51E-05

BHE81_19020	Sugar ABC transporter ATP-binding protein	4.02	7.72E-05
Genes considered differentially expressed with a $\|log_2(\text{fold change, FC})\| \geq 1.5$ and adjusted *p*-value (p-adj) ≤ 0.01 detected at least three methods, log_2FC and p-adj values were calculated by edgeR (See Materials and Methods for details).			

Table S9. Fatty acid composition of strain AqSCr in the presence and absence (controls) of 11 mM Cr(VI)

FAMES	LB (pH 8) with 11mM Cr(VI)	LB (pH 8)	2way ANOVA *p*-value
$C_{12:0}$	0.4±0.3	0.8±0.2	0.9575
$C_{14:1}$	0.2±0.0	0.2±0.0	>0,9999
$C_{14:0}$	6.5±0.8	8.9±0.3	<0,0001
$C_{16:1}$	21.3±0.6	27.8±0.5	<0,0001
$C_{16:0}$	26.5±0.2	23.8±0.2	<0,0001
$C_{17:1}$	0.4±0.0	0.6±0.2	>0,9999
$C_{18:1}$	26.1±0.8	24.1±0.3	<0,0001
$C_{18:0}$	0.6±0.1	0.4±0.1	0.9989
Hydroxy FAMEs			
α-OH $C_{12:0}$	0.6±0.0	0.1±0.1	0.6877
β-OH $C_{14:0}$	9.6±0.4	8.5±0.3	0.0016

β-OH $C_{16:0}$	0.1±0.1	0.1±0.0	>0,9999

REFERENCES

1. D. F. Ackerley, C. F. Gonzalez, C. H. Park, R. Blake 2nd, M. Keyhan, and A. Matin, Chromate-reducing properties of soluble flavoproteins from *Pseudomonas putida* and *Escherichia coli*, *Appl. Environ. Microbiol.*, 2004, **70**, 873-882. doi: 10.1128/AEM.70.2.873-882.2004.
2. H. Jin, Y. Zhang, G. W. Buchko, S. M. Varnum, H. Robinson, T. C. Squier and P. E. Long, Structure determination and functional analysis of a chromate reductase from *Gluconacetobacter hansenii*, *PLoS One*, 2012, **7**, e42432. doi: 10.1371/journal.pone.0042432.
3. Y. H. Kwak, D. S. Lee and H. B. Kim, *Vibrio harveyi* nitroreductase is also a chromate reductase, *Appl. Environ. Microbiol.*, 2003, **69**, 4390-4395. doi: 10.1128/AEM.69.8.4390-4395.2003.
4. J. Mazoch, R. Tesarík, V. Sedlácek, I. Kucera and J. Turánek, Isolation and biochemical characterization of two soluble iron(III) reductases from *Paracoccus denitrificans*, *Eur. J. Biochem.*, 2004, **271**, 553-562. doi: 10.1046/j.1432-1033.2003.03957.x
5. A. Morokutti, A. Lyskowski, S. Sollner, E. Pointner, T. B. Fitzpatrick, C. Kratky, K. Gruber and P. Macheroux, Structure and function of YcnD from *Bacillus subtilis*, a flavin-containing oxidoreductase, *Biochemistry*, 2005, **44**, 13724-13733. doi: 10.1021/bi0510835
6. D. J. Opperman and E. van Heerden, A membrane-associated protein with Cr(VI)-reducing activity from *Thermus scotoductus* SA-01, *FEMS Microbiol. Lett.*, 2008, **280**, 210-218. doi: 10.1111/j.1574-6968.2007.01063.x
7. D. J. Opperman, L. A. Piater and E. van Heerden, A novel chromate reductase from *Thermus scotoductus* SA-01 related to old yellow enzyme, *J. Bacteriol.*, 2008, **190**, 3076-3082. doi: 10.1128/JB.01766-07.
8. C. H. Park, M. Keyhan, B. Wielinga, S. Fendorf and A. Matin, Purification to homogeneity and characterization of a novel *Pseudomonas putida* chromate reductase, Appl. Environ. Microbiol., 2000, **66**, 1788-1795. doi:10.1128/AEM.66.5.1788-1795.2000.
9. K. J. Robins, D. O. Hooks, B. H. Rehm and D. F. Ackerley, *Escherichia coli* NemA is an efficient chromate reductase that can be biologically immobilized to provide a cell free system for remediation of hexavalent chromium, *PLoS One*, 2013, **8**, e59200. doi: 10.1371/journal.pone.0059200.
10. X. M. Xue, Y. Yan, H. J. Xu, N. Wang, X. Zhang and J. Ye, ArsH from *Synechocystis* sp. PCC 6803 reduces chromate and ferric iron, *FEMS Microbiol. Lett.*, 2014, **356**, 105-112. doi: 10.1111/1574-6968.12481.
